# Extensive field evidence for the release of HONO from the photolysis of nitrate aerosols

**DOI:** 10.1126/sciadv.add6266

**Published:** 2023-01-18

**Authors:** Simone T. Andersen, Lucy J. Carpenter, Chris Reed, James D. Lee, Rosie Chance, Tomás Sherwen, Adam R. Vaughan, Jordan Stewart, Pete M. Edwards, William J. Bloss, Roberto Sommariva, Leigh R. Crilley, Graeme J. Nott, Luis Neves, Katie Read, Dwayne E. Heard, Paul W. Seakins, Lisa K. Whalley, Graham A. Boustead, Lauren T. Fleming, Daniel Stone, Khanneh Wadinga Fomba

**Affiliations:** ^1^ Wolfson Atmospheric Chemistry Laboratories, Department of Chemistry, University of York, York, UK.; ^2^ FAAM Airborne Laboratory, Cranfield, UK.; ^3^ National Centre for Atmospheric Science, University of York, York, UK.; ^4^ School of Geography, Earth and Environmental Sciences, University of Birmingham, Birmingham, UK.; ^5^ Instituto Nacional de Meteorologia e Geofísica, São Vicente (INMG), Mindelo, Cabo Verde.; ^6^ School of Chemistry, University of Leeds, Leeds, UK.; ^7^ Atmospheric Chemistry Department, Leibniz Institute for Tropospheric Research (TROPOS), Leipzig, Germany.

## Abstract

Particulate nitrate (${\mathrm{pNO}}_{3}^{-}$) has long been considered a permanent sink for NO*_x_* (NO and NO_2_), removing a gaseous pollutant that is central to air quality and that influences the global self-cleansing capacity of the atmosphere. Evidence is emerging that photolysis of ${\mathrm{pNO}}_{3}^{-}$ can recycle HONO and NO*_x_* back to the gas phase with potentially important implications for tropospheric ozone and OH budgets; however, there are substantial discrepancies in “renoxification” photolysis rate constants. Using aircraft and ground-based HONO observations in the remote Atlantic troposphere, we show evidence for renoxification occurring on mixed marine aerosols with an efficiency that increases with relative humidity and decreases with the concentration of ${\mathrm{pNO}}_{3}^{-}$, thus largely reconciling the very large discrepancies in renoxification photolysis rate constants found across multiple laboratory and field studies. Active release of HONO from aerosol has important implications for atmospheric oxidants such as OH and O_3_ in both polluted and clean environments.

## Teaser

Aircraft observations in the remote Atlantic troposphere show evidence for HONO production occurring on various aerosol types.

## INTRODUCTION

Nitrous acid (HONO) has a pivotal role in tropospheric chemistry as an important source of the hydroxyl radical (OH) (*1*–*7*). It has also been proposed as a substantial source of NO*_x_* (NO and NO_2_) to the remote marine environment (*8*–*10*). NO*_x_* regulates the abundance of atmospheric oxidants and is essential for the formation of secondary atmospheric aerosols, and OH controls the self-cleansing capacity of the atmosphere via degradation of pollutants and greenhouse gases such as methane (*1*).

HONO is produced through the gas-phase reaction of NO and OH radicals (R1) and lost through photolysis (R2), reaction with OH radicals (R3), and dry deposition (R4)

$$\mathrm{NO}+\mathrm{OH}+M\;\overset{k_{1}}{\to }\;\mathrm{HONO}+M$$ (R1)

$$\mathrm{HONO}+hv(<390\;\mathrm{nm})\;\overset{j\mathrm{HONO}}{\to }\;\mathrm{OH}+\mathrm{NO}$$ (R2)

$$\mathrm{HONO}+\mathrm{OH}\;\overset{k_{3}}{\to }\;H_{2}O+{\mathrm{NO}}_{2}$$ (R3)

$$\mathrm{HONO}\;\overset{k_{\mathrm{dep}}}{\to }$$ (R4)

In the remote oceanic troposphere, NO*_x_* levels are too low to supply any notable levels of HONO from R1 and primary HONO emission sources such as vehicle exhaust, wild fires, and soils (*2*, *3*, *11*) are absent. Recent measurements also suggest the absence of an ocean surface source (*8*). Reactions on aerosol surfaces have historically been suggested to make only moderate contributions to daytime HONO formation (*5*), although there is evidence for NO_2_-to-HONO conversion on aerosols in polluted to semi-polluted regions (*4*, *6*, *7*). However, recent field observations in the marine atmosphere have indicated that photolysis of particulate nitrate (${\mathrm{pNO}}_{3}^{-}$) associated with sea salt aerosol can be an important source of HONO and NO*_x_* (*9*, *10*, *12*), consistent with laboratory studies demonstrating that photolysis of ${\mathrm{pNO}}_{3}^{-}$ is significantly enhanced compared to photolysis of gaseous HNO_3_, with HONO and NO*_x_* as the major products (*13*–*20*)

$${\mathrm{pNO}}_{3}^{-}+hv\overset{j{\mathrm{pNO}}_{3}^{-}}{\to }x\mathrm{HONO}+y{\mathrm{NO}}_{2}$$ (R5)

This “renoxification” process is important because it offers a rapid route for recycling of NO*_x_* from inorganic nitrate, which has historically been thought to be slow because of the small photolysis frequency of gas phase HNO_3_. If renoxification supplies a substantial amount of NO*_x_* to remote oceanic regions, where sources have been considered to be limited primarily to ship emissions and to transport and decomposition of peroxyacetyl nitrate, it could have a global-scale impact on production of tropospheric oxidants such as O_3_ and OH and, hence, on methane removal (*21*).

The photolysis rate constant of renoxification, *j*${\mathrm{pNO}}_{3}^{-}$, is typically expressed as a ratio to the gas-phase HNO_3_ photolysis frequency, giving an enhancement factor *f* = $\frac{j{\mathrm{pNO}}_{3}^{-}}{j{\mathrm{HNO}}_{3}}$. There is a very high uncertainty in *f*, with laboratory and field studies reporting values spanning three orders of magnitude (Table 1). Field observations of HONO in the remote oceanic atmosphere, which offer a robust method to diagnose the presence of any missing sources but are so far limited to only a few days of measurements, have been reconciled with known sources and sinks using *f* of between ~25 and 450 (*9*, *12*, *21*, *22*). This range is within reported values from laboratory studies on various surfaces (*14*, *18*–*20*, *23*) and aerosol filter samples (*17*) of between ~10 and 1700. However, recent experiments using suspended nitrate particles (*23*) and calculations derived from observed ratios of NO*_x_*/HNO_3_ in the polluted boundary layer (*24*) have derived a much smaller *f* of 1 to 30. Thus, there is as yet no consensus on whether renoxification offers a limited or a highly significant role in the NO*_x_* and OH budgets of remote environments or field evidence for HONO production from renoxification occurring on ambient aerosol other than sea salt aerosol.

**Table 1.** Overview of previous studies investigating the production of HONO from surface-adsorbed HNO_3_/nitrate. Concentrations of ${\mathrm{pNO}}_{3}^{-}$ given in other units than mol m^−3^ were converted using temperature at 298 K and pressure at 1 atm. ${j\mathrm{pNO}}_{3}^{-}$ was determined from the production of HONO + NO_2_, and ${j\mathrm{pNO}}_{3}^{-}\to \mathrm{HONO}$ is determined from the production of HONO. Enhancement factors were estimated using *j*HNO_3_ = 7 × 10^−7^ s^−1^ if not reported in the study.

Study type	Surface	[pNO_3_^−^] (10^−9^ mol m^−3^)	*j*pNO_3_^−^ (10^−5^ s^−1^)	*j*pNO_3_^−^ → HONO (10^−5^ s^−1^)	Enhancement factor (*f*)	Airmass origin	Reference
Field	Aerosols	0.04–2.0		20	150–450	Marine	(*9*)
Field/laboratory	Aerosols	0.7–39.5	0.62–50.0		8–700	Urban, rural, remote	(*17*)
Field/model	Aerosols	40–265*			1–30		(*24*)
Global model	Aerosols	0.4–40			25–100	Marine	(*21*)
Laboratory	Aerosols†	1600–9700			<10		(*23*)
Field/laboratory	Aerosols	5–15			18–54‡	Marine	(*12*)
	**Surface**	**Density of HNO**_**3**_ **on surface (10**^**−7**^ **mol m**^**−2**^**)**	***j*****pNO**_**3**_^−^ **(10**^**−5**^ **s**^**−1**^**)**	***j*****pNO**_**3**_^−^ → **HONO (10**^**−5**^ **s**^**−1**^**)**	**Enhancement factor (** ***f*** **)**	**Airmass origin**	
Laboratory	Urban Grime		120		1700	Urban	(*14*)
Laboratory	Aluminum	4–251	5.7–15.3		8–220		(*20*)
Laboratory	Oak	3–174	1.6–37.0		20–530		(*20*)
Laboratory	Maple	22–380	0.9–4.9		10–70		(*20*)

*${\mathrm{pNO}}_{3}^{-}$ here is the sum of ${\mathrm{pNO}}_{3}^{-}$ and HNO_3_ (5th to 95th percentiles).

†Pure nitrate salts added to Teflon chambers.

‡Twenty-fifth to75th percentiles.

## RESULTS

**Fig. 1.**
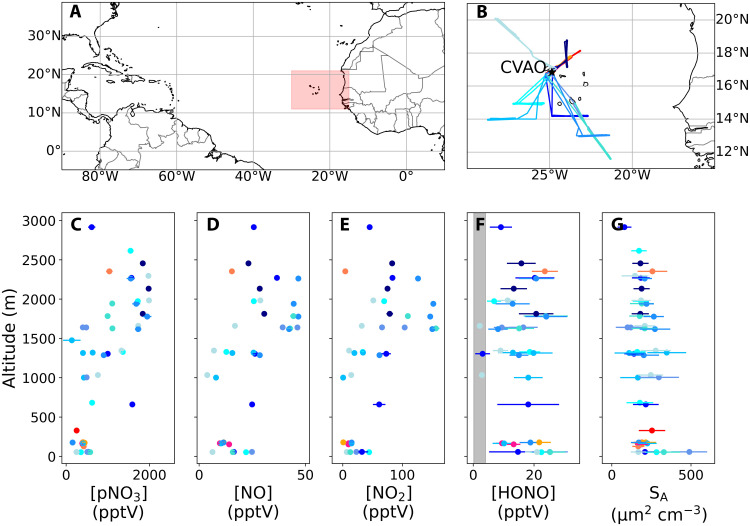
Flight tracks and vertical profiles of $p{\mathrm{NO}}_{3}^{-}$, NO, NO_2_, HONO, and aerosol surface area during ARNA-1 (August 2019) and ARNA-2 (February 2020). (**A**) Map over the region, where the red box is the area shown in (B). (**B**) Flight tracks from ARNA-1 in red colors and ARNA-2 in blue colors, alongside the location of the CVAO. The vertical profiles of (**C**) ${\mathrm{pNO}}_{3}^{-}$, (**D**) NO, (**E**), NO_2_, (**F**) HONO, and (**G**) aerosol surface area are colored by their respective flight tracks as shown in (B). Each data point is an average of an SLR. The gray vertical line in (F) shows the range of calculated HONO gas-phase source–only concentrations for each SLR during the flights. The error bars represent the 1σ uncertainties described in Supplementary Text.

The ARNA (Atmospheric Reactive Nitrogen over the remote Atlantic) field campaigns took place over the tropical Atlantic Ocean in August 2019 and February 2020 using the FAAM BAe-146-301 atmospheric research aircraft and in August 2019 at the Cape Verde Atmospheric Observatory (CVAO; Fig. 1A). Twelve flights (four in summer and eight in winter; Fig. 1B) were conducted with in situ measurements including NO, NO_2_, HONO, O_3_, and aerosol surface area. ${\mathrm{pNO}}_{3}^{-}$ was determined from aerosol filters sampled over each straight-and-level run (SLR). Photolysis rates and OH radical concentrations were modeled using the global three-dimensional atmospheric chemistry model GEOS-Chem. At the CVAO, NO*_x_*, ${\mathrm{pNO}}_{3}^{-}$, O_3_, and photolysis rates are measured routinely, and these were supplemented by HONO measurements during the ARNA campaigns (*10*).

**Fig. 2.**
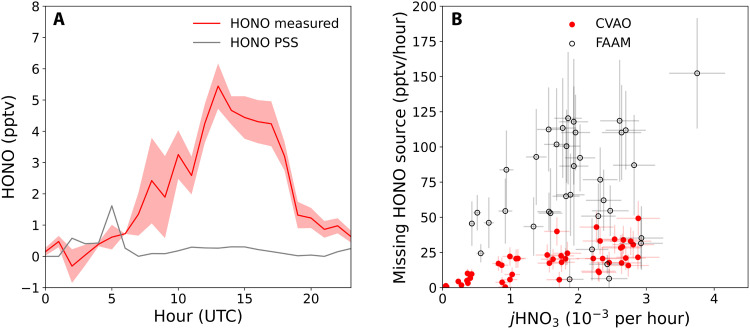
Evidence for a photochemical origin of the missing HONO source. (**A**) Average diurnal cycle of HONO mixing ratios measured at the CVAO in August 2019 (red), where the shaded area shows ± the SE of the measurements, compared to calculated HONO photostationary state (PSS; see Supplementary Text) mixing ratios (gray). The deviation from PSS maximizes around local noon, indicating that the additional source term has a strong photolytic dependence. (**B**) Missing HONO source at the CVAO (hourly averages, red circles, *r*^2^ of linear fit = 0.79) and for the aircraft data (hourly averages, black unfilled circles, *r*^2^ of linear fit = 0.06) plotted against *j*HNO_3_.

Figure 1 (C to G) shows the aircraft vertical profiles of NO, NO_2_, HONO, and ${\mathrm{pNO}}_{3}^{-}$ and total aerosol surface area. For NO, NO_2_, and ${\mathrm{pNO}}_{3}^{-}$, the vertical profiles show clear enhancements between 1500 and 2500 m. The air sampled in this layer predominately originates from over Africa (figs. S1 and S2) and shows tracers of biomass burning and dust in the aerosol composition (figs. S3 and S4). The mean mixing ratios of HONO (±1 SD) were 18.2 ± 5.9 parts per trillion by volume (pptv) in the marine boundary layer (MBL) and 14.2 ± 6.4 pptv above the MBL (0.5 to 3.0 km). Figure 2A shows that HONO levels measured at the CVAO were about a factor of 3 lower (4.7 ± 1.8 pptv at solar noon) than the MBL aircraft measurements but similar to previous measurements made at the CVAO (~3.5 pptv at solar noon) (*10*).

The lifetime of HONO during the ARNA and CVAO campaigns was approximately 12 min; thus, the steady-state HONO concentrations can be estimated from balancing the known in situ production and loss mechanisms described in R1 to R4 (see the Supplementary Materials). For the entire dataset, measured HONO levels were substantially larger than these calculated levels, which are negligible in this very low NO*_x_* environment (Figs. 1F and 2A), demonstrating the presence of an additional source. Figure 2B shows that the missing HONO source required to balance the measured HONO concentrations was strongly correlated with solar irradiance (plotted as *j*HNO_3_) in the CVAO data (*r*^2^ of linear fit = 0.79), consistent with a photochemical mechanism such as reaction R5. For the CVAO campaign, HONO was measured over full diurnal cycles (i.e., a large range of solar irradiance values) in homogeneous air experiencing relatively invariant ${\mathrm{pNO}}_{3}^{-}$concentrations (mean and SD of 19.0 ± 5.6 nmol m^−3^). The aircraft sorties experienced a much larger range of ${\mathrm{pNO}}_{3}^{-}$ concentrations (33.5 ± 19.8 nmol m^−3^), aerosol compositions, and relative humidities (RHs), and here, the relationship between the missing HONO source and *j*HNO_3_ was weak. Potential reasons for the lower missing HONO source in the ground-based CVAO measurements are discussed below.

**Fig. 3.**
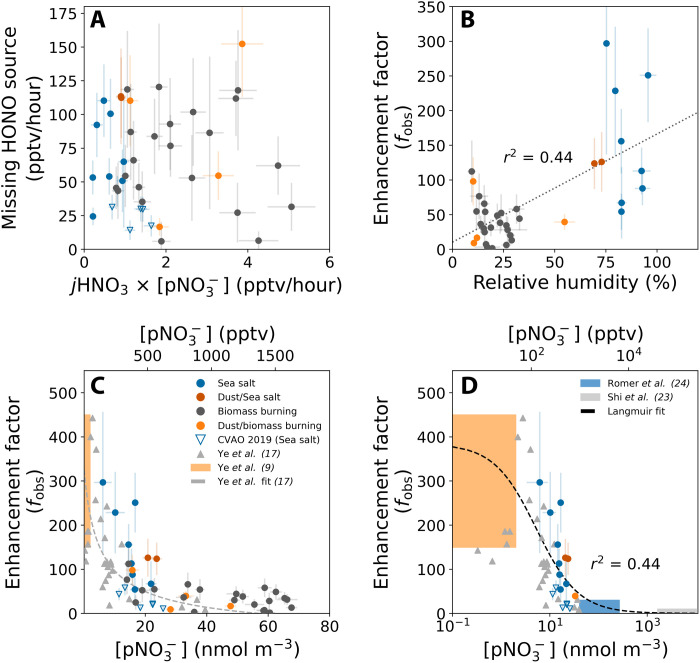
Relationships of *P*_HONOhet_ and enhancement factors with various atmospheric parameters. (**A**) Calculated missing HONO source (*P*_HONO_het__) versus the product of ${\left[{\mathrm{pNO}}_{3}^{-}\right]}_{\mathrm{bulk}}$ and the photolysis frequency of gaseous HNO_3_. Note the lack of a simple linear relationship. Data points are colored according to the dominant aerosol classification (Supplementary Materials). ${\left[{\mathrm{pNO}}_{3}^{-}\right]}_{\mathrm{bulk}}$ was converted from nmol m^−3^ to pptv using surface pressure and temperature. The ${\left[{\mathrm{pNO}}_{3}^{-}\right]}_{\mathrm{bulk}}$ used is a lower limit, meaning the derived *f* are upper limits, due to a potentially lower than 100% sampling efficiency of coarse mode aerosols (>1 μm) for aircraft measurements (*56*–*58*). (**B**) Derived enhancement factors as a function of RH. (**C**) Derived enhancement factors as a function of ${\left[{\mathrm{pNO}}_{3}^{-}\right]}_{\mathrm{bulk}}$. Also shown are the laboratory data of Ye *et al.* (*17*) using aerosol filters (gray triangles) and their fit to the data (gray dashed line) and the aircraft data of Ye *et al.* (*9*) from the remote North Atlantic marine boundary layer (orange rectangle). (**D**) Enhancement factors as a function of a wide range of ${\left[{\mathrm{pNO}}_{3}^{-}\right]}_{\mathrm{bulk}}$. Same as (C) but a wider comparison of *f*_obs_ with previously reported values and data restricted to conditions where the aerosol was expected to be deliquesced. The dotted line shows the Langmuir fit (Eq. 4) applied to all data.

The missing HONO source is plotted against *j*${\mathrm{HNO}}_{3}\times [{\mathrm{pNO}}_{3}^{-}]$ in Fig. 3A. If the missing source was entirely due to renoxification (*P*_HONO_het__), then it should be equal to the product of ${j\mathrm{pNO}}_{3}^{-}\;\mathrm{and}\;{\mathrm{pNO}}_{3}^{-}$ and the slope equal to *f* because *f* = $\frac{{j\mathrm{pNO}}_{3}^{-}}{{j\mathrm{HNO}}_{3}}$. Figure 3A shows, however, that there was no simple linear relationship between these parameters, unlike a previous field study of renoxification occurring on sea salt aerosol in the marine atmosphere (*9*). These results indicate that more factors were influencing ${j\mathrm{pNO}}_{3}^{-}$ than simply the intensity of solar radiation.

The ARNA flights encompassed a range of RH, from low humidities (<~30% RH), where sea salt particles are expected to be dry and solid, to those where they become deliquesced (>45 to 55% RH) and therefore contain some water on the surface, to high humidities (>75% RH), where sea salt aerosols will be present as suspended droplets (*25*). We find significant differences in *f* across these RH ranges, with average values of 156 ± 91 (1σ), 96 ± 49, and 39 ± 29 for >75% RH, 53 to 75% RH, and <30% RH, respectively (*P* < 0.0001 when comparing the samples where RH >75% and RH <30%). Figure 3B illustrates the general tendency for *f* to increase with RH; a simple linear fit has an *r*^2^ of 0.44. Solid-phase nitrate aerosol exhibits a much lower quantum yield of photolysis compared to aqueous phase (*26*) because the products cannot diffuse out of the particle but instead will recombine. A marked increase in *f* on particulate sodium nitrate between dry conditions (~3% RH), with negligible renoxification, and higher RH representing aqueous aerosol have been found in the laboratory (*23*). Our observations show that such effects are identifiable in the atmosphere.

We also find that *f* reduces with $\left[{\mathrm{pNO}}_{3}^{-}\right]$, as shown in Fig. 3C, which shows both our aircraft and ground-based (CVAO) data. In air masses dominated by sea salt aerosol with generally low [${\mathrm{pNO}}_{3}^{-}$], we find an average *f* calculated from the aircraft data of 157 (range, 54 to 297), similar to the airborne study of Ye *et al.* (*9*) who derived *f*_obs_ of 150 to 450 on sea salt aerosol. We observed similar enhancement factors for mixed sea salt/dust aerosol; however, significantly lower *f* was derived for biomass burning aerosol, which contained high $\left[{\mathrm{pNO}}_{3}^{-}\right]$ (*f* of ~10 to 60). Laboratory measurements of HONO production from photolysis of aerosol filter samples collected in urban, suburban/rural, and remote areas (*17*) reported a notably similar relationship between the enhancement factor and $\left[{\mathrm{pNO}}_{3}^{-}\;\right]$ as our study. The Ye *et al.* (*17*) fit to their laboratory data is shown in Fig. 3C as the gray dashed line. Their ${j\mathrm{pNO}}_{3_{\mathrm{effective}}}^{-}$ was converted to an enhancement factor using *j*HNO_3_ = 7 × 10^−7^ s^−1^, which corresponds to typical tropical summer conditions on the ground (solar elevation angle θ = 0°) as simulated in their light-exposure experiments, thus

$$f=\frac{\frac{6.1\times 10^{-4}\times \ln(1+4.4\times 10^{-1}\times [{\mathrm{pNO}}_{3}^{-}])}{\left[{\mathrm{pNO}}_{3}^{-}\right]}-3.5\times 10^{-5}}{7\times 10^{-7}}$$ (1)

where [${\mathrm{pNO}}_{3}^{-}$] is the ambient concentration in units of 10^−9^ mol m^−3^ (air). This empirical relationship was attributed by Ye *et al.* (*17*) to a surface catalysis mechanism for renoxification because it is consistent with that observed in the photolysis of surface-adsorbed HNO_3_/nitrate on various surfaces (*20*). Our observations span a much wider range of ${\mathrm{pNO}}_{3}^{-}$ concentrations than previous field studies (*9*, *10*, *12*), allowing the relationship of $\left[{\mathrm{pNO}}_{3}^{-}\right]$ with the renoxification rate constant to be explored in the field. Given that the Ye *et al.* (*17*) experiments were carried out on aerosol collected primarily from urban or urban-influenced locations, it is not unexpected to find significant differences in the absolute values of *f* between and within these studies. However, Fig. 3C demonstrates a remarkable similarity in the general relationship of *f* with bulk nitrate.

Recent laboratory experiments and field observations of NO*_x_*/HNO_3_ ratios have derived enhancement factors of <30 and have suggested therefore that renoxification plays only a limited role in atmospheric chemistry (blue and gray boxes in Fig. 3D) (*23*, *24*). However, these studies were carried out under very high ${\mathrm{pNO}}_{3}^{-}$ mass concentrations, where our observations would suggest only low enhancement factors that have only a small dependence on ${\mathrm{pNO}}_{3}^{-}$ across the concentration ranges explored.

**Fig. 4.**
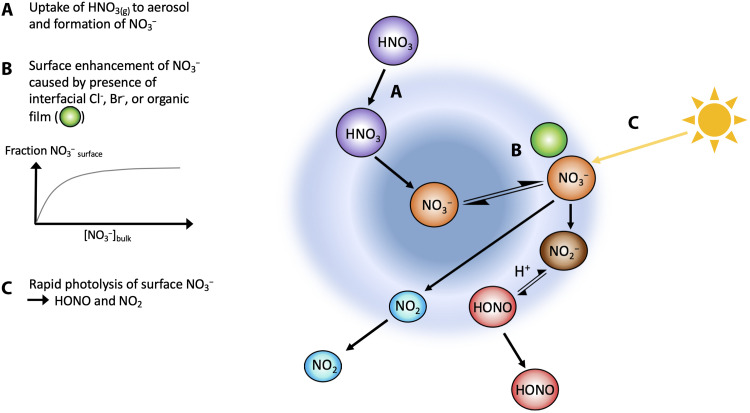
Potential surface-mediated mechanism for renoxification on nitrate aerosol. Nitrate photolysis can be enhanced by the presence of halide ions, which have a well-established affinity for the surface. This has been attributed to the enhanced partitioning of nitrate ions at the interface because of the existence of a double layer of interfacial halide ions and subsurface counterion (*31*–*35*).

We next further explore the relationship between *f* and [${\mathrm{pNO}}_{3}^{-}$]. A number of studies have indicated that renoxification is driven by photolysis of surface-bound rather than bulk nitrate. Nitrate or nitric acid adsorbed on surfaces can undergo a much more rapid photolysis compared to bulk aqueous nitrate or gas phase HNO_3_ due to enhanced absorption cross sections arising from optimal alignment and orientation of HNO_3_ molecules on surfaces (*13*, *15*, *27**)* and high quantum yields (compared to the aqueous phase) due to reduced solvent cage effects (*13*). Therefore, if the nitric acid is located substantially on or near the aerosol surface, its photolysis rate could be enhanced by orders of magnitude over that of gas-phase nitric acid. While molecular dynamics simulations offer conflicting results as to whether nitrate anions prefer interfacial to bulk solvation, the presence of certain cations has been shown experimentally to lead to preferential distributions of nitrate ion at the interface (*28*–*30*). In addition, several experimental studies show that the products of nitrate photolysis are enhanced by the presence of halide ions, a phenomenon that has been attributed to the known surface affinity of halide ions pulling sodium cations closer, in turn, drawing NO_3_^−^ to the interface (*31*–*35*). We illustrate this surface-enhanced mechanism in Fig. 4. Potential synergisms between HNO_3_ and organic films (*36*, *37*) could further enhance the concentration and photochemistry of surface nitrate compared to bulk aerosol nitrate.

If renoxification is controlled by the availability of surface ${\mathrm{NO}}_{3}^{-}$, then we would expect *f* to be dependent on the partitioning between the equilibrium surface and equilibrium bulk nitrate in liquid (deliquesced) aerosol. If nitrate behaves as a surfactant, as suggested by the theoretical and laboratory studies discussed above, then such partitioning can be described using a Langmuir adsorption isotherm

$${\left[{\mathrm{pNO}}_{3}^{-}\right]}_{\mathrm{surface}}=\frac{Q^{0}\;K_{L}\;{\left[{\mathrm{pNO}}_{3}^{-}\right]}_{\mathrm{bulk}}}{1+K_{L}{\left[{\mathrm{pNO}}_{3}^{-}\right]}_{\mathrm{bulk}}}$$ (2)

where *Q*^0^ is the maximum loading of adsorbate ${\mathrm{NO}}_{3}^{-}$ corresponding to complete monolayer coverage [reaching saturation under conditions of high solute ([${\mathrm{pNO}}_{3}^{-}$]_bulk_) concentration] and *K*_L_ is the Langmuir equilibrium constant of $\${NO}_{3}^{-}\$$.

Because we measured bulk $\${\mathrm{pNO}}_{3}^{-}\$$ rather than surface-bound, we can define *P*_HONO_het__ as $j{\mathrm{pNO}}_{3_{\mathrm{effective}}}^{-}\times {\left[{\mathrm{pNO}}_{3}^{-}\right]}_{\mathrm{bulk}}$ and the observationally derived enhancement factor, *f*_obs_, as $\frac{{j\mathrm{pNO}}_{3_{\mathrm{effective}}}^{-}}{{j\mathrm{HNO}}_{3}}$. Thus, it follows that

$$f_{\mathrm{obs}}=\frac{{j\mathrm{pNO}}_{3_{\mathrm{surface}}}^{-}}{{j\mathrm{HNO}}_{3}}\times \frac{{\left[{\mathrm{pNO}}_{3}^{-}\right]}_{\mathrm{surface}}}{{\left[{\mathrm{pNO}}_{3}^{-}\right]}_{\mathrm{bulk}}}$$ (3)

The dependence of *f*_obs_ on [${\mathrm{pNO}}_{3}^{-}$]_bulk_ can then be estimated by combining Eqs. 2 and 3

$$f_{\mathrm{obs}}=\frac{f.{\left[{\mathrm{pNO}}_{3}^{-}\right]}_{\mathrm{surface}}}{{\left[{\mathrm{pNO}}_{3}^{-}\right]}_{\mathrm{bulk}}}=\frac{f.Q^{0}\;K_{L}}{1+K_{L}{\left[{\mathrm{pNO}}_{3}^{-}\right]}_{\mathrm{bulk}}}$$ (4)

where *f =* ${j\mathrm{pNO}}_{3_{\mathrm{surface}}}$/*j*HNO_3_.

Figure 3D shows how the Langmuir expression (Eq. 4) fits the enhancement factors derived from our field data under conditions where the aerosol was expected to be deliquesced (RH > 53%; colored circle and empty blue triangles), along with the Ye *et al.* (*9*) marine boundary layer data (orange box), the laboratory data of Ye *et al.* (*17*) (gray symbols) and of Shi *et al.* (*23*) (gray bar), and those derived from ambient measurements of the NO*_x_*/HNO_3_ ratio (blue bar) (*24*).

The best fit for Eq. 4 was found, using the mean *f* of 70 and [${\mathrm{pNO}}_{3}^{-}$]_bulk_ concentrations in units of 10^−9^ mol m^−3^ air (as determined from filter measurements), with *Q*^0^ = 29 nmol m^−3^ and *K*_L_ = 0.19 nmol^−1^ m^3^. Calculations using the aerosol thermodynamics module ISORROPIA II (*38*) using the ARNA aerosol composition, temperature, and humidity data (see the Supplementary Materials) show that for RH > 53%, essentially all (>99%) of the nitrate (gas phase HNO_3_ and ${\mathrm{pNO}}_{3}^{-}$) was in the particle phase. For equivalently humid conditions, the solute ([${\mathrm{pNO}}_{3}^{-}$]_bulk_) concentrations are thus proportional to the [${\mathrm{pNO}}_{3}^{-}$] concentrations in air, and we use the latter for ease of comparison across studies. Note that Eq. 4 only considers production of HONO and ignores any coproduction of NO*_x_*. This is important to note when comparing our enhancement factors to laboratory studies, which have measured the production of all gaseous oxidized nitrogen products although is of limited consequence if the yield of HONO is >0.9, as suggested from a budget analysis of field measurements (*22*).

Although there is significant variance in the data around the simple Langmuir fit of *f*, likely due to differences in humidity and aerosol composition as discussed above, the reasonable fit (*r*^2^ = 0.44) demonstrates a potential explanation for the observed dependence of the renoxification enhancement factor on ${\left[{\mathrm{pNO}}_{3}^{-}\right]}_{\mathrm{bulk}}$ across multiple studies. Alternative mechanisms for these observed relationships include competing decomposition pathways of NO_2_^−^ leading to greater conversion to non-HONO products at high nitrate concentrations (*39*) and a reduction in the quantum yield for NO_2_^−^ formation at high nitrate concentrations (*40*). Noting that halide anions and sodium cations have been suggested to lead to enhancement of the surface concentration and photochemistry of surface nitrate compared to bulk aerosol nitrate, we also plot *f* against [pCl^−^]/[p${\mathrm{NO}}_{3}^{-}$] and [pNa^+^]/[p${\mathrm{NO}}_{3}^{-}$] in fig. S5. While the enhancement factor generally increases with these ratios, this could simply be a reflection of the relationship of *f* with RH (Fig. 3B) because RH was higher in air masses containing elevated sea salt. Ultimately, our field observations are not able to demonstrate what factors besides RH and [${\mathrm{pNO}}_{3}^{-}$] control the renoxification efficiency but suggest that additional variables are affecting *f* to an important degree.

## DISCUSSION

The observations shown here represent the most extensive field measurements of HONO in the marine atmosphere to date and confirm its widespread presence in the marine lower troposphere at mixing ratios in the range of ~5 to 20 pptv. The fact that HONO production occurs in association with a wide range of aerosol types shows that renoxification is not limited to sea salt aerosol although is strongly restricted under dry conditions (such as those in the free troposphere), where aerosols are not deliquesced.

Nevertheless, the relationships found between the enhancement factor *f* and aerosol composition, alongside the fact that the derived ${\mathrm{pNO}}_{3}^{-}$ photolysis rates are orders of magnitude higher than gas phase nitric acid photolysis rates, are consistent with a surface aerosol mechanism for renoxification. The observed reduction in the renoxification efficiency with increasing concentration of ${\mathrm{pNO}}_{3}^{-}$ helps reconcile the very large discrepancies in renoxification photolysis rate constants found across multiple laboratory and field studies. The observations emphasize the importance in laboratory renoxification studies of generating aerosol composition as representative as possible of the clean marine troposphere.

The relationships observed among *f*, humidity, and [${\mathrm{pNO}}_{3}^{-}$] were undoubtedly affected by additional sources of variability. In sunlight, NO_3_^−^ photolysis occurs predominantly via two channels: one producing the nitrite anion (NO_2_^−^) and O(^3^P) and the other producing NO_2_ and OH (*39*). Gaseous HONO production from the former pathway requires acidity and has been found to be strongly dependent on aerosol pH (*16*). Here, we note that the ground-based CVAO HONO measurements were associated with lower enhancements (shown in Fig. 3C) compared to the aircraft observations. One potential reason for this is that the surface measurements experience fresh rather than aged sea salt aerosol due to sampling within the surf zone. They would therefore likely be associated with higher aerosol pH, which disfavors HONO production. It has also been shown that the renoxification rate constant can be enhanced in the presence of organic matter, through various mechanisms (*16*, *24*, *36*, *41*–*45*). It is expected therefore to find a variation of *f* beyond that described by our simple relationships, although investigation of these additional factors is outside the scope of our study.

Because it has been previously determined that *f* of 25 to 50 occurring only on sea salt aerosol results in peak enhancements of 20 to 60% for OH, 10 to 30% for ozone, and up to a factor of 20 for NO*_x_* concentrations in the tropical and subtropical marine boundary layer (*21*), it is clear that the rapid production of HONO observed in this study occurring on diverse aerosol types, showing a mean *f* of 70 across all samples, will have a substantial impact on our understanding of atmospheric oxidant cycling. We note additionally that nitrate aerosols have become increasingly more important in the atmosphere because of an increase in precursor ammonia emissions and a decline of ammonium sulfate aerosols (*46*). Thus, recycling of nitric acid to nitrogen oxides on nitrate aerosol could have important, increasing, and as yet unexplored implications for the trends and distributions of atmospheric oxidants.

## MATERIALS AND METHODS

At the CVAO, HONO was measured using a long-path absorption photometer (LOPAP-03, QUMA GmbH) (*47*). The calibration and standard operating procedures are described by Kleffman and Wiesen (*48*). In 2019 (data shown in Fig 2), the instrument was deployed on top of a 7.5-m tower, and the detection limit was 1.1 pptv (2σ, 30 s). Because of the sampling time for the aerosol composition being 24 hours, the CVAO HONO data used in Fig. 3 were averaged over 1000 to 1500 local time to illustrate their relationship with ${\mathrm{pNO}}_{3}^{-}$. Aerosol samples collected at the CVAO were analyzed for major ions using a standard ion chromatography technique as described by Fomba *et al.* (*49*). Filters were changed every 24 hours, and the composition was assumed to be uniform across the sampling period. NO*_x_* has been measured continually at the CVAO since 2006 using a chemiluminescence instrument (Air Quality Design Inc.) (*50*, *51*).

On the FAAM aircraft, NO*_x_* (NO + NO_2_) and HONO were measured using differential photolysis (*52*), where NO_2_ and HONO are photolytically converted into NO, followed by NO chemiluminescence detection using a dual-channel instrument equipped with two custom-built photolytic converters. The HONO conversion efficiencies were calibrated against an ultraviolet-visible cavity enhanced absorption spectroscopy using HIRAC [The Highly Instrumented Reactor for Atmospheric Chemistry (*53*)] similarly to Reed *et al.* (*52*) (see the Supplementary Materials). The average HONO detection limit over the SLR was 4.2 pptv (2σ). Aerosol chemical composition was determined by off-line analysis of filter samples. Two identical inlets are mounted on the port side of the aircraft allowing collection of duplicate samples. During the ARNA campaigns, sampled aerosol were divided into two size fractions according to aerodynamic particle diameter (approximately corresponding to >1 and <1 μm). Filters were stored frozen (−20°C) until extraction. Anions (Cl^−^, NO_3_^−^, NO_2_^−^, SO_4_^2−^, and C_2_O_4_^2−^) and cations (Na^+^, K^+^, NH_4_^+^, Ca^2+^, and Mg^2+^) were determined in the aqueous extracts using ion chromatography (Thermo Fisher Scientific, Dionex-1100; see the Supplementary Materials).

Photolysis rates and OH concentrations were extracted for all ground and airborne observations at the nearest point in space and time from the GEOS-Chem model (v12.9.0, DOI:10.5281/zenodo.3950327). The model was run at a nested horizontal resolution of 0.25° × 0.3125° over the region (−32.0° to 15.0°E, 0.0° to 34.0°N), with boundary conditions provided by a separate global model run spun up for 1 year. The photolysis rates are calculated online in quadrature using Fast-JX code (*54*, *55*). Comparison with available campaign observations is described in the Supplementary Materials.

## Supplementary Materials

### This PDF file includes:

Supplementary Text Figs. S1 to S9 Table S1 References

## Notes

View/request a protocol for this paper from *Bio-protocol*.
