# Supplementary material for: Extensive field evidence for the release of HONO from the photolysis of nitrate aerosols
Source: Sci Adv. 2023 Jan 18;9(3):eadd6266. doi: 10.1126/sciadv.add6266 (PMC9848427; doi:10.1126/sciadv.add6266)
Supplement: Supplementary file 1 — Supplementary Text Figs. S1 to S9 Table S1 References [file sciadv.add6266_sm.pdf]

Science Advances
AAAS

Supplementary Materials for

Extensive field evidence for the release of HONO from the photolysis of nitrate aerosols

Simone T. Andersen *et al.*

Corresponding author: Lucy J. Carpenter, lucy.carpenter@york.ac.uk; Simone T. Andersen, simone.andersen@york.ac.uk.

Sci. Adv. **9**, eadd6266 (2023)
DOI: 10.1126/sciadv.add6266

This PDF file includes:

Supplementary Text
Figs. S1 to S9
Table S1
References

Location

The Cape Verde Atmospheric Observatory (CVAO; 16° 51' N, 24° 52' W) is located on the north eastern coast of the island of São Vicente, Cabo Verde. The air masses arriving at the CVAO predominantly come from the northeast (>95% of all wind direction measurements) and have travelled over the Atlantic Ocean for multiple days since their last exposure to anthropogenic emissions, with the potential exception of ship emissions (*59, 60*). The origin of the air masses arriving at the CVAO have been shown to be very diverse; North America, the Atlantic, Europe, Arctic, and African regions (*51*). During the spring and summer, the air masses predominantly originate from the Atlantic, while during the winter the CVAO receives air mainly from the Sahara, resulting in very high wintertime dust loadings (*49, 61, 62*). The time zone of Cabo Verde is UTC-1. A full description of the CVAO site and associated measurements is given in Carpenter et al. (*59*).

The FAAM airborne laboratory is a modified BAe-146-301 4-engine jet equipped with atmospheric measurement instruments to measure a range of gas-phase species, aerosol composition and size distribution, and meteorological parameters. Four flights north-east of the CVAO were conducted during the ARNA-1 campaign (August 19th-20th 2019). During ARNA-2 (February 5th-12th 2020), flights were targeted on locations where both dust and biomass burning outflow were predicted to be present by 5-day within forecast model predictions, by the NASA GEOS-CF (*63*) and GEOS-5 models respectively (see flight tracks in Fig. 1). Multiple straight-and-level-runs (SLRs) of ~20 minutes were carried out on all flights, at altitudes between 100 ft and 10,000 ft, and the analyses in this study are focused on these data.

1 CVAO Measurements

Measurements of NO_x, HONO and the composition of aerosols at the CVAO have been described in detail elsewhere (*10, 49, 50*) so only a brief description is given here. NO_x has been measured continually since 2006 using a chemiluminescence instrument (Air Quality Design Inc.) (*50, 51*); hourly averages are available alongside the rest of the continuous CVAO data at the Norwegian Institute for Air Research (NILU) EBAS database. The NO_x uncertainties are described in Andersen et al. (*50*).

HONO was measured using a Long Path Absorption Photometer (LOPAP-03, Quma GmbH) during three campaigns; November-December 2015 (reported in Reed et al. (*10*)), August 2019, and February 2020. HONO was sampled within a stripping coil into an acidic solution and derivatized with an azo dye. Absorption of light (550 nm) by the azo dye was measured with an Ocean Optics spectrometer using an optical path length of 2.4 m. The relative error of the LOPAP is estimated at 10%. The technique is described in detail in Heland et al. (*47*) and the calibration and standard operating procedures are described in Kleffman and Wiesen. (*48*).

In 2015 the instrument was deployed in the CVAO guest lab sampling at a height of ~3 m and the detection limit was 0.2 ppt (2 sigma, 30 seconds), as described in Reed et al. (*10*). The same location was used in 2020 and the detection limit was 0.7 pptv. In 2019, the instrument was deployed on top of the 7.5 m tower and the detection limit was 1.1 pptv (2 sigma, 30 seconds). Only measurements from 2019 are used here due to the other two campaigns sampling at low heights, with potential surface and/or enhanced surf zone effects. Fig. 3 shows the average diurnal profile of the HONO measurements during August 2019. Due to the sampling time for the aerosol composition being 24 hours, the HONO measurements were averaged daily using the midday (11.00-16.00 UTC) measurements.

Aerosol samples have been collected at the CVAO since 2007 and analysed for Na^+, NH_4^+, K^+, Mg^{2+}, Ca^{2+}, Cl^-, Br^-, NO_3^-, SO_4^{2-} and $C_2O_4^{2-}$ using a standard ion-chromatography technique as described in Fomba et al. (*49*). The detection limits for all ions

as measured by the conductivity detection technique were less than 0.002 µg m^{-3} except for calcium, which was 0.02 µg m^{-3}. Filters were changed every 24 hours during campaigns and the composition was assumed to be uniform across the sampling period.

The solar actinic UV flux was measured using a spectral radiometer (a 2-pi sr quartz diffuser coupled to an Ocean Optics QE65000 spectrometer via a 10 m fibre optic cable) giving photolysis rates for a variety of species. Photolysis rates were also modelled using GEOS-Chem as explained in section 4. A comparison between the measured and modelled photolysis rates is shown in Fig. S6. Here we use modelled values throughout to avoid discarding data with missing measured photolysis rates. The uncertainty in the modelled photolysis rates was estimated as the average difference between the modelled and measured photolysis rates, as discussed in section 3.1.

2 FAAM Measurements

2.1 NO_x and HONO

NO_x ($NO + NO_2$) and HONO were measured using differential photolysis (*52*), where NO_2 and HONO are photolytically converted into NO (R6 and R2) followed by NO chemiluminescence detection (R7-R8).

$NO_2 + h\nu \rightarrow NO + O(^3P)$	($h\nu \leq 410$ nm)	(R6)
$HONO + h\nu \rightarrow NO + OH$	($h\nu < 390$nm)	(R2)
$NO + O_3 \rightarrow NO_2^* + O_2$		(R7)
$NO_2^* \rightarrow NO_2 + h\nu$	($h\nu > 600$ nm)	(R8)

A dual-channel NO_x chemiluminescence instrument equipped with two custom-built photolytic converters was used, similar in design to that described in Pollack et al. (*64*). Each converter consists of a ~ 40 cm^3 quartz cylinder with two external light emitting diodes (LEDs) to avoid heating up the sampled air and causing an NO_2 artefact (*50, 65*). Channel 1 and 2 are fitted with 385 nm LEDs (Hamamatsu, jNO_2 1.3 s^{-1}) to optimize the NO_2 conversion and 365 nm LEDs (Hamamatsu, jNO_2 1.0 s^{-1}) to optimize HONO conversion, respectively. Channel 1 switches between zero and NO_x+HONO measurements and channel 2 switches between zero, NO, and NO_x+HONO (see Fig. S7). Zero measurements are performed to determine the signal due to dark current and interferences. Calibration sequences were conducted multiple times during each flight to determine the NO sensitivity, NO_2 conversion efficiencies, and potential offsets between the two channels. Standard addition of approximately 5 ppbv NO was used to calibrate the sensitivity and conversion efficiencies, where the offset was determined as the difference in measured ambient NO mixing ratio, when running both channels in NO mode at the same time. The sensitivity and conversion efficiencies for both channels were stable during each flight making interpolation between each calibration appropriate.

The NO mixing ratio was determined from the NO measurements on channel 2 using the in-flight determined sensitivity. The NO_2 mixing ratio was therefore estimated from the NO_x+HONO measurements on channel 1 by subtracting the signal due to NO (from channel 2) and the measured offset in the ambient NO measurements between the two channels using the in-flight sensitivity and NO_2 conversion efficiency of channel 1. The HONO mixing ratio was determined from the difference between the two channels when the LEDs are on using equation (IV), where $\mathrm{NO}_{2_{365}}^{\dagger}$ and $\mathrm{NO}_{2_{385}}^{\dagger}$ are the NO_2 mixing ratio of each channel if the entire signal was due to NO_2 and $\mathrm{CE}_{365}^{\mathrm{HONO}}$ and $\mathrm{CE}_{365}^{\mathrm{HONO}}$ are the conversion efficiencies of HONO for each channel (*52*).

$$[\mathrm{HONO}] = \frac{\mathrm{NO}^{\dagger}_{2_{365}} - \mathrm{NO}^{\dagger}_{2_{385}}}{\mathrm{CE}^{\mathrm{HONO}}_{365} - \mathrm{CE}^{\mathrm{HONO}}_{385}} \quad \text{(IV)}$$

The HONO conversion efficiencies are dependent on the NO_2 conversion efficiencies, so $\mathrm{CE}^{\mathrm{HONO}}_{365} - \mathrm{CE}^{\mathrm{HONO}}_{385}$ was calibrated against UV-vis Cavity Enhanced Absorption Spectroscopy (UV-CEAS) using HIRAC (The Highly Instrumented Reactor for Atmospheric Chemistry (*53*)) similarly to Reed et al. (*52*). HONO was introduced into the HIRAC chamber from a photolytic source described in Boustead (*66*). A humidified mixture of NO in N_2 was illuminated by a low-pressure mercury vapour lamp emitting at 185 nm. Photolysis of H_2O in the presence of O_2 produces OH and HO_2, which react with NO forming HONO, NO_2, and OH.

$H_2O + h\nu \rightarrow OH + H$ ($h\nu$ =185 nm) (R9)
$H + O_2 \rightarrow HO_2$ (R10)
$OH + NO \rightarrow HONO$ (R11)
$HO_2 + NO \rightarrow NO_2 + OH$ (R12)

In addition, unreacted NO remains. A constant flow of HONO in nitrogen was added to the chamber at a rate of approximately 1 L min^{-1} along with a separate flow of nitrogen to balance the sampling rates of the connected instruments to ensure the chamber pressure remained constant at 1000 mbar. This resulted in a gradual increase over time in the HONO concentration present in the chamber. The HONO concentration was monitored by UV-CEAS. The optical cavity was aligned across the diameter of the chamber positioned directly next to the sampling location of the differential photolysis instrument. The CEAS instrument consisted of a probe light produced by a Laser Driven Light Source (LDLS—Energetiq EQ-99X) producing near constant radiance from the NIR to the UV, <200 nm. The light was then directed into the chamber where the cavity was generated between two cavity mirrors (99.2-99.7% from 330-370 nm). Light exiting the cavity was focused into a fibre optic connected to the detector, a high throughput spectrograph (CP140-103, f/2) coupled to a fast-read (1 kHz) line-array CCD (Hamamatsu S7031).

To prevent saturation of the detector at wavelengths outside the region of interest, a 450 nm cut off filter was used to remove the longer wavelengths and a cuvette filled with acetone removed the peaks in the far UV, <250 nm. Measurements were taken at 30 second intervals. Data were analysed between the 330 nm and 370 nm, with the absorption spectrum plotted against the literature cross section. The path length was determined separately by measuring the absorption spectrum of a known concentration of NO_2.

The sensitivity of the FAAM NO_x instrument to HONO was derived from a linear fit ($R^2 = 0.85$, Slope/$\mathrm{CE}^{\mathrm{HONO}}_{365} - \mathrm{CE}^{\mathrm{HONO}}_{385}$ = 0.775) of UV-CEAS-derived [HONO] against differential-derived [HONO].

2.1.1 Uncertainty Analysis

An extensive uncertainty analysis for NO, NO_2 and HONO was performed for each SLR during the two airborne campaigns. The precision of the measurements was determined from the zero count variability of each SLR which is directly related to the photon-counting precision of the PMT (*67*):

$$\text{Zero count variability} = x - \bar{x} \quad \text{(V)}$$

where x is the individual zero measurements during a run, and $\bar{x}$ is the mean of the measurements on the same run. The 1σ precision of NO, $NO^{\dagger}_{2_{365}}$, and $NO^{\dagger}_{2_{385}}$ were on average 0.6 pptv, 1.2 pptv, and 1.1 pptv for the SLRs, respectively, using equation (VI):

$$\text{Precision} = \frac{1\sigma}{\sqrt{\text{number of averaging points}}} \quad \text{(VI)}$$

where σ is the standard deviation of the zero count variability, which is converted into a mixing ratio using the sensitivity and conversion efficiency of each channel. The 1σ precision of HONO was determined to on average be 2.1 pptv by propagating the precision of $NO^{\dagger}_{2_{365}}$ and $NO^{\dagger}_{2_{385}}$ and taking the calibrated HONO conversion into account.

The calibration uncertainty consists of the uncertainties in the sensitivity (S), the drift in sensitivity (S_{drift}), the conversion efficiency (CE), drift in conversion efficiency (CE_{drift}), and the flow (C_{flow}) and concentration (C_{conc}) of the calibration gas (equation (VII) and (VIII)). Since the HONO measurements were calibrated on the ground, the uncertainty is estimated from those measurements. The calibration uncertainty of NO, $NO^{\dagger}_{2_{365}}$, $NO^{\dagger}_{2_{385}}$, and HONO were found to be on average 2.4%, 2.6%, 2.8%, and 15%, respectively.

$$\text{NO cal uncertainty} = \sqrt{S^2 + S^2_{drift} + C^2_{flow} + C^2_{conc}} \quad \text{(VII)}$$

$$NO_2 \text{ cal uncertainty} = \sqrt{S^2 + S^2_{drift} + C^2_{flow} + C^2_{conc} + CE^2 + CE^2_{drift}} \quad \text{(VIII)}$$

The total absolute uncertainties of NO, NO_2, and HONO were determined by propagating the appropriate measurement precisions and uncertainties in the calibration and drift in measurements using the rules for addition, subtraction, multiplication, and division. The total 1σ uncertainties were on average 0.8 pptv, 3.5 pptv, and 4.4 pptv, for NO, NO_2, and HONO, respectively for the SLRs.

2.2 Aerosol Composition

2.2.1 Sampling

Aerosol chemical composition was determined by off-line analysis of filter samples. Two identical inlets are mounted on the port side of the aircraft allowing collection of duplicate samples. Air was pumped from the inlets through stacks of filter substrates contained within housings on the inside of the aircraft. The total air flow through the sample lines was monitored by mass flow meters.

During the ARNA campaigns stacks of two 47 mm diameter filters of different pore size were used; a 1 µm filter was placed at the bottom of the stack (ARNA-1: Whatman, PTFE with polypropylene mesh back; ARNA-2: Whatman, polycarbonate, Nuclepore Track-Etch membrane) and an 8 µm filter (Whatman, polycarbonate, Nuclepore Track-Etch membrane, 47mm diameter) at the top, so the aerosols were divided into two size fractions according to the filter pore size (> 8 µm and 1-8 µm). These fractions broadly correspond to 'coarse' (> 1 µm aerodynamic diameter) and 'fine' (< 1 µm aerodynamic diameter) aerosol (*56, 57*). To minimise sample contamination, filter holders were washed in deionised water, loaded with filters, stacked together and wrapped in a clean polythene bag pre-flight, and only unwrapped immediately before use. Prior to each flight, the sampling lines were cleaned with deionised water to remove remnant material from the previous flights. Duplicate samples were collected during the flights by inserting a stack of filters into each sample line, zeroing the

mass flow meters, turning on the pump and opening the air flow. Three sets of duplicate samples were taken during each of the four ARNA-1 flights. Usually, two sets of filters were deployed in the boundary layer and a set in the free troposphere. During ARNA-2 a single stack of filters were sampled on each SLR and duplicates were only collected on days with one flight. At least once a day a set of blank samples was also collected by inserting the stack of filters into the sampling line without turning the air flow on. During ARNA-1 blank samples were collected during one of the free troposphere runs, while during ARNA-2 blank samples were collected either on the way to the sampling region or when returning to São Vicente. Post-flight, the filters were removed from the units and inserted into sterile lab tubes (CORNING Centristar, polypropylene, 50 mL (ARNA-1) and 15 mL (ARNA-2)) and kept cold (5°C) to minimise the aerosols evaporating off the filters. Upon return to the laboratory, filters were stored frozen (-20°C) until extraction.

2.2.2 Analysis

Filters were extracted by adding 3 mL ultrapure water (≥18.2 MΩ cm^{-1}) to the filter in the polypropylene storage tube then ultra-sonicating (Fisher scientific, FB15051) for 3 × 10 min, with one minute of vortex mixing (SciQuip) after every 10 min of sonication. Using a needle and syringe, the aqueous extract was aspirated from the tube and passed through a syringe filter (Milex, 0.22 µm pore size) into a second, pre-cleaned tube (15 mL, CORNING Centristar, polypropylene, cleaned with ultrapure water).

Anions (Cl^-, NO_2^-, NO_3^-, Br^-, SO_4^{2-}, and $C_2O_4^{2-}$) and cations (Na^+, K^+, NH_4^+, Ca^{2+}, Mg^{2+}) were determined in the aqueous extracts using ion chromatography (IC; Thermo Fischer, Dionex-1100) with isocratic elution and 0.1 mL injection volume. The anions and cations were separated using Dionex IonPac AS14A and CS12A columns, respectively. Eluent was prepared from ultrapure water with Na_2CO_3, and $NaHCO_3$ for anions and methane sulfonic acid for cations. Calibration standards were prepared daily using salts of the relevant ions (Analytical Reagent grade or better) and ultrapure water. Standards contained between 0 µM and 500 µM of each anion and between 0 µM and 40 (500 for Na^+) µM for each cation. 0.5 mL aliquots of samples and calibration standards were pipetted into polyvials (Thermo Scientific, 0.5 mL) and capped with a plain polyvial cap before loading into the IC autosampler. The samples were run on the IC immediately following extraction.

Each sample was corrected for procedural contamination using the blank samples collected on each flight. Blank corrected extract concentrations below the analytical limit of detection (LOD) were substituted with 0.75 × LOD as described by Chance et al. (*68*) before being converted to aerosol loadings using the extraction solvent volume and the air volume passed through each filter. The LOD was calculated as the median of the daily determined analytical LODs from the calibration curves. Aerosol ion concentrations from ARNA-1 and ARNA-2 are compared to previous measurements in the same region in Table S1. All the measurements are in the same range as the previous measurements except NH_4^+, which is higher than previously reported. For the winter samples this could be due to the air being sampled having significant contributions from biomass burning, which is a known source of NH_4^+, K^+, and NO_3^- (*69, 70*).

Sea-salt and non-sea-salt (NSS) components were calculated from the seawater ratios between sodium and other ions (*71*), assuming that all measured sodium was from sea-salt. The measured concentrations of anions and cations for each individual sample are plotted in Fig. S3 and S4, respectively. Previous aerosol measurements made at the CVAO have shown that NSS Ca^{2+} varied from 0.01-4.44 ug m^{-3} over a 5 year period with the maximum concentrations corresponding to Saharan dust events and the minimum concentrations to clean marine air (*49*).

2.2.3 Uncertainty Analysis

The total uncertainty in each ion was estimated by propagation of the uncertainties in the blanks, the calibration curve, and the air volume. The uncertainty in the blanks was estimated as the standard deviation of all the blanks for each size fraction. The calibration uncertainty was determined as the 1σ confidence interval of the calibration curve by the Chromeleon 7 software. The uncertainty in the air volume sampled has previously been estimated as 0.5 L for total sample volumes up to 400 standard litre (stL) for the BAe-146 system (*72*), however, as the sample volumes measured in this study where significantly higher (900-5000 stL) the uncertainty was set conservatively to 1% of the air volume. The uncertainties associated with each ion are plotted as error bars in Fig. S3 and S4.

$$u_{\mathrm{Sample-Blank}}(\mu\mathrm{M}) = \sqrt{u_{\mathrm{Calibration}}^2 + u_{\mathrm{Blank}}^2} \qquad \text{(IX)}$$

$$u_{\mathrm{conc}}\,(\%) = \sqrt{\left(\frac{u_{\mathrm{Sample-Blank}}}{\mathrm{Sample-Blank}}\right)^2 + (u_{\mathrm{Airflow}})^2} \qquad \text{(X)}$$

Additional uncertainty is associated with the sampling efficiency of the inlet lines. Andreae et al. (*57*) judged the sampling efficiency to be high for fine aerosols of a similar system on another aircraft, but only ~35% for coarse aerosols. Sanchez-Marroquin et al. (*58*) characterized the inlet system on the BAe-146, where again fine aerosols had a high sampling efficiency but the sampling efficiency of coarse mode aerosols depended on their diameter. However, as the effect has not been quantified it has not been included in the plotted uncertainties.

2.3 Aerosol Surface Area

In situ measurements of aerosol particle concentration distributions were made with the Passive Cavity Aerosol Spectrometer Probe (PCASP) and the Cloud Droplet Probe (CDP), both manufactured by Droplet Measurement Technologies (DMT). These instruments are both laser scattering based optical particle counters, operating at 632.8 nm and 658 nm for the PCASP and CDP respectively. Nominal particle size ranges are 0.1-3 μm and 3-50 μm and a composite particle size distribution was constructed using the two instruments to cover the entire size range. Both instruments had a sample rate of 1 Hz. They were mounted on underwing pylons, the CDP is open path while the PCASP uses a pump and very short inlet. The same CDP was used for both campaigns while different PCASPs were used due to changes in instrument serviceability.

The instruments were calibrated as described by Rosenberg et al. (*73*). The CDP was cleaned and calibrated throughout the campaigns on every flying day. An average calibration was calculated for each campaign and applied to all flights in that campaign. The PCASP was calibrated in the laboratory before or after the campaigns with each calibration applied to all flights within the campaign. The sample flow rate through the PCASP was calibrated with a Gilibrator-2 Calibrator (Sensidyne). The size calibration determines the range of scattering cross-sections associated with each of the thirty bins of both instruments. For computational simplicity, spherical particles were assumed so that Mie theory could be used to calculate equivalent particle diameters. The predominant aerosol type was identified as described in section 6 for each run. Size calibrations were calculated for three different types of aerosol runs; runs in sea salt aerosol, runs in mineral dust, and runs in a combination of biomass burning aerosol and dust. Despite any mix of particle type in a single run, the most appropriate size calibration was applied to all particles in that run.

Sea salt dominated runs were at altitudes less than 300 m above the sea surface. The OPAC database (*74*, *75*) includes the optical properties for both accumulation and coarse mode sea salt aerosols as a function of scattering wavelength and relative humidity. Relative

humidity for the sea salt runs was determined from the GEOS-chem model as 79% ± 3% so the OPAC data for 80% relative humidity was used for all sea salt runs. The accumulation mode refractive index applied to the PCASP calibrations and that of the coarse mode, applied to the CDP calibrations, were both 1.35+2.0e-8i.

The optical properties of aged mineral dust transported from the sub-Saharan region have been measured at the ground station in Cabo Verde and during previous aircraft campaigns based in the region. Ryder et al. (*76*) lists such campaigns since 2006. Here we use a range of size-invariant refractive indices based on previous measurements. Weinzierl et al. (*77*) use a non-absorbing refractive index for particles larger than 3 µm, however, this has not been done here. Ryder et al. (*78*) use values for the real and imaginary parts of the refractive index ranging over 1.53-1.55 and 0.001-0.0024i and we use values based on these. The real part is biased a little smaller than the 1.55 (all reported values have been corrected for the wavelengths used here) from airborne measurements reported by Weinzierl et al. (*77*) and from ground-based electron microscopy measurements by Kandler. (*79*). A sensitivity study in Ryder et al. (*76*) suggest that a ± 0.05 change will result in a change in derived effective diameter of < 5% and so has only a small influence on the derived particle sizes. Weinzierl et al. (*77*) reported imaginary refractive indices of 0.0014 and 0.001 for 632.8 and 658 nm for sizes less than 2.5 um. Ryder et al. (*76*) used a constant 0.001. Ground-based measurements at the CVAO suggest somewhat larger values of 0.0026 and 0.0025 (*80*). Again sensitivity studies for values from 0 to 0.006 suggest changes in effective diameter of between 1 % (*77*) and 5 % (*76*) depending on the condition of the study.

Uncertainties in the refractive index have been included in the bin size calibrations (*73*) by calculating the bin diameters from the bin scattering cross-sections over the range of values, 1.53-1.55 and 0.001-0.0024i. The resulting uncertainties of bin centre and widths are larger than for a single value refractive index and propagated through to the derived property uncertainties. Similarly to studies quoted above, this additional uncertainty has a minor impact on the run-averaged sizes and their uncertainties.

Transported biomass burning aerosols with a strongly absorbing soot content were assumed to be concentrated in the small size range (*81, 82*). A strong accumulation mode, not seen in either the mineral dust or sea salt runs, was a feature of the area concentration distributions when sampling biomass burning plumes. A two-part calibration was applied in this case (*81*); for particles nominally smaller than 300 nm a biomass specific refractive index of 1.57 + 0.043i was used while for larger particles the mineral dust refractive index was used (*77*).

Uncertainties of the PCASP measurements were derived from the counting statistics and an assumed 10% uncertainty in the sample flow rate. The impact of uncertainties of the externally measured ambient conditions was found to be small so errors in the ambient pressure and temperature were ignored. The gain stage for the smallest particles of the PCASP used during ARNA-1 failed between the campaign and the post-campaign calibration. The calibration of this gain stage was taken from a subsequent calibration and used for these bins, the uncertainties associated with these bins was doubled as a precaution.

The sample area of the CDP was determined by the manufacturer using a droplet gun as described by Lance et al. (*83*), no uncertainties were given so 20% has been assumed here. The collection optics operated over solid angles subtended by 1.7-14 deg. Counting statistics were again included in the propagated errors and uncertainties in the measured true air speed were omitted.

To obtain particle surface area concentrations over the entire size range sampled a composite number concentration distribution was determined. Firstly the bins either side of the gain stage cross-overs of the PCASP were merged (*73*) and the first bin for both instruments discarded due to uncertainty of the lower bound of the first bins. Any

overlapping bins were resampled to match the CDP bins and an average, taken weighted by the associated uncertainties in the number concentration. For particles at the upper limits of the PCASP measurement range, uncertainties of PCASP number concentrations were significantly larger than those of the CDP at these sizes. Bin centre diameters were used to calculate the particle surface areas assuming spherical particles. The bin width added in quadrature with the error in the centre and width, determined the uncertainty in the particle diameter and this was propagated through to the calculated bulk properties.

For each run, outliers of the bulk properties with a z-score of >=5 were discarded as questionable, less than 1 % of the data of any run were discarded. Run averages were calculated weighted by the uncertainties of the bulk parameters in each 1 s sample. The uncertainties of the run averages are thus a combination of the propagated uncertainties of the 1 s data and the natural variability along each run. These two are uncorrelated and so were added in quadrature to obtain a final uncertainty for each bulk parameter calculated. Except for cases with very low counts and so large counting errors, the along-run variability tended to dominate the uncertainty calculated.

The effective diameter, d_{eff} (or surface mean diameter in Hinds (*84*)), over the entire size range measured tend to be larger than those reported by Weinzierl et al. (*77*) but for mineral dust are comparable with those of Ryder et al. (*78*) There is little correlation with altitude. Limiting the bulk parameters to the accumulation mode, here those approximately less than 2.5 µm, the average of all d_{eff} for dust, sea salt, and biomass burning runs were 0.59 ± 0.09, 0.39 ± 0.12, and 0.25 ± 0.18 µm respectively.

2.4 Photolysis Rates

Photolysis rates were measured on the aircraft using two spectral radiometers (a 2-pi sr quartz diffuser coupled to an Ocean Optics QEPro spectrometer via a fibre optic cable); one upward facing and one downward facing to measure the direct solar actinic UV flux and the scattered light. The total photolysis rate is determined as the sum of the two measurements.

3 Modelling – GEOS-Chem

Photolysis rates and OH concentrations were extracted for all ground and airborne observations at nearest point in space and time from the GEOS-Chem model (v12.9.0, DOI:10.5281/zenodo.3950327). The model was run at a nested horizontal resolution of 0.25x0.3125 degrees over the region (-32.0 to 15.0 °E, 0.0 to 34.0 °N), with boundary conditions provided by a separate global model run spun up for one year. The photolysis rates are calculated online in quadrature using Fast-JX code (*54, 55*).

3.1 Uncertainty Analysis

A comparison between measured and modelled jHONO and $j\mathrm{HNO_3}$ photolysis rates during ARNA-1 is shown in Fig. S8. The uncertainty in the modelled photolysis rates is determined as the difference between the modelled and measured rates for a SLR, which on average was 15 and 11% for jHONO and $j\mathrm{HNO_3}$, respectively.

GEOS-Chem modelled OH concentrations have previously been compared to observations and been shown to be simulated to within observational uncertainty (74% to 135%, 2σ confidence interval) (*85*). As the OH reactions with HONO and NO are minor contributions towards the calculated missing HONO source, the OH uncertainty makes a negligible contribution to the overall uncertainty, however, here we use 37% (1σ) for all further uncertainty analysis.

4 Trajectory Analysis

For each aerosol sample, 96-hour back trajectories were modelled along the flight track using the Hybrid Single-Particle Lagrangian Integrated Trajectory (HYSPLIT) model (*86*). Seven out of 8 air samples taken during ARNA-1 travelled over the Atlantic Ocean during the 4 days before being sampled compared to 3 out of 38 samples taken during ARNA-2 (see Figs. S1 and S2). Modelled precipitation along the back trajectories was used to evaluate whether aerosols could have been rained out before reaching the aircraft. Precipitation was only observed for the ARNA-1 back trajectories.

5 Aerosol Classification

The aerosol samples were divided into 5 categories based on their composition (Fig. S3 and S4), back trajectories (Fig. S1 and S2) and concurrent gas-phase measurements:

- Dust: Fomba et al. (*49*) observed NSS Ca^{2+} (PM_{10}) to vary from 0.01-4.44 ug m^{-3} from 2007-2011 at the CVAO and they associated the highest NSS Ca^{2+} measurements with dust episodes. Therefore, we categorised samples as dust if the back trajectories crossed the Saharan desert in the previous 96 hours and contained >4 ug m^{-3} (99.75 nmol m^{-3}) NSS Ca^{2+}. It should also be noted that the two observed dust samples were also high in sea-salt due to being sampled at low altitude (see back trajectories).
- Sea-salt: These samples contained high concentrations of sea-salt (Na^{+}, Cl^{-}) and low concentrations of NSS Ca^{2+}, NSS K^{+}, and NSS Mg^{2+}. The back trajectories were either completely over the Atlantic Ocean (e.g. ARNA-1 except filter 5) or close to the ocean for an extended amount of time previous to being sampled (e.g. filter 9 and 14).
- Free troposphere: Sample 31 was observed to be low in all anions and cations and the back trajectories associated with this sample were above 1000 m in altitude for the 96 hours before sampling, suggesting that the free troposphere was sampled.
- Biomass burning (BB): The statistical threshold approach described by Lee et al. (*87*) was used to determine whether the sampling took place in a biomass burning plume based on CO, O_3, and HCN measurements. The percentage of each SLR spent in BB is plotted in Fig. S9. The gas-phase BB filter was combined with the measured composition of the aerosols. Biomass burning releases potassium and nitrate to the atmosphere, and so the ratios of these species to other aerosol constituents may be used as tracers. Elevated ratios of NSS K^{+} to NSS Ca^{2+} in fine mode (<1 μm) aerosol have been associated with biomass burning (*69*). Similarly, the NO_3^{-}/NSS SO_4^{2-} ratio in aerosols measured at Barbados from the trade winds from Africa has been reported to be 0.4 during the summer, where the pollution is dominated by fossil fuel combustion in Europe and 1.4 during the winter, where the pollution is dominated by wood and biomass burning in Africa (*88*). Here, we considered samples to be influenced by biomass burning when the NSS K^{+}/NSS Ca^{2+} ratio of the fine fraction was above a threshold value of 0.24 (derived from a crustal K^{+}/Ca^{2+} of ~0.71 (*89*) and water solubility of 24% for K^{+} and 71% for Ca^{2+} in Saharan dust aerosol [data available via GEOTRACES IDP2021 from (*90*)]), and the NO_3^{-} to NSS SO_4^{2-} ratio was above 1.4.
- Dust/Biomass burning: The remaining samples were categorised as a mixture of dust and biomass burning.

6 Calculation of gas-phase only $[HONO]_{PSS}$

The steady-state levels of observed [HONO], $[HONO]_{PSS}$, (grey area in Figure 1F) were calculated using equation (XI):

$$[\mathrm{HONO}]_{\mathrm{PSS}} = \frac{k_1[\mathrm{OH}][\mathrm{NO}]}{k_3[\mathrm{OH}]+j\mathrm{HONO}+k_{\mathrm{dep}}} \quad \text{(XI)}$$

where k_1 and k_3 are the rate coefficients for the reaction of OH radicals with NO and HONO, respectively, taken from Atkinson et al. (*91*), and jHONO is the modelled photolysis rate of HONO. HONO deposition is assumed to be negligible for the aircraft measurements. For calculating $[\mathrm{HONO}]_{\mathrm{PSS}}$ at the CVAO we calculate k_{dep} using a value of 3 cm s^{-1} for the HONO deposition velocity (*92-95*) divided by the effective boundary layer height *h*, which is the maximum height where dry deposition is still relevant. *h* was determined to vary from 175-440 m using the average Deardorff velocity (D_v) (*96*) and the calculated HONO photolysis lifetimes (τ_{HONO}). The average Deardorff velocity measured during SLRs at ~100 ft in August 2019 (ARNA-1) was 0.3 m s^{-1}.

7 Missing HONO Source

Recent studies in sub-polluted and polluted environments have proposed NO_2 uptake on illuminated aerosols to be an additional source of daytime HONO (*7, 43, 97-99*). Here we evaluate this process as a source of HONO in this study. Assuming that all NO_2 taken up on aerosols converts into HONO with a 100% yield, the HONO production rate from NO_2 uptake on aerosols can be determined using equation (XII), where *k* is the reaction rate coefficient for NO_2 uptake described by equation (XIII) (*43*). $\gamma_{\mathrm{NO_2\rightarrow HONO}}$ is the reactive uptake coefficient of NO_2 to generate HONO, SA is the total surface area of the aerosols sampled, and *v* is the mean thermal velocity of NO_2.

$$-\frac{\mathrm{d}[\mathrm{NO_2}]}{\mathrm{d}t} = \frac{\mathrm{d}[\mathrm{HONO}]}{\mathrm{d}t} = k[\mathrm{NO_2}] \quad \text{(XII)}$$

$$k = \frac{\gamma_{\mathrm{NO_2\rightarrow HONO}} \times \mathrm{SA} \times v}{4} \quad \text{(XIII)}$$

Dyson et al. (*43*) found $\gamma_{\mathrm{NO_2\rightarrow HONO}}$ on TiO_2 aerosols to depend on relative humidity and the initial NO_2 mixing ratio with the highest uptake coefficients measured at 25-30% relative humidity and approximately $[NO_2]$ = 50 ppbv. $\gamma_{\mathrm{NO_2\rightarrow HONO}}$ was shown to drop from 1.26×10^{-4} to approximately 4×10^{-5} when going from 50 ppbv to 34 ppbv of initial NO_2. These values are in good agreement with the initial uptake coefficients of NO_2 reported by Li et al. (*100*) for mineral dust, however, the steady-state uptake coefficients by Li et al. (*100*) were significantly lower. To evaluate an upper limit of the HONO production by NO_2 uptake on aerosols, a γ of 10^{-4} is used for sea-salt and dust and 10^{-5} is used for biomass burning/soot (*43, 100*). Using the maximum observed values for total aerosol surface area and NO_2 concentrations, the HONO production rate from NO_2 uptake on aerosols is estimated to be less than 1 pptv h^{-1} for all three types of aerosols, making it negligible for the conditions of this study.

The missing HONO source required to supply the levels of observed [HONO] is, therefore, assumed to be entirely due to photolysis of particulate nitrate and estimated assuming steady state from equation (XIV):

$$P_{HONO_{\mathrm{het}}} = k_3[\mathrm{OH}][\mathrm{HONO}] + j\mathrm{HONO}[\mathrm{HONO}] + k_{\mathrm{dep}}[\mathrm{HONO}] - k_1[\mathrm{OH}][\mathrm{NO}]$$
(XIV)

The uncertainty in the missing HONO source is determined by calculating the relative uncertainty in each term using the propagation of errors rule for multiplication. The relative

uncertainties are then converted into absolute uncertainties, which are propagated according to the rule of addition and subtraction to give final average uncertainty of 47%.

8 Enhancement Factor: Uncertainty Analysis

The enhancement factor, f_{obs}, was estimated from equation (XV):

$$f_{obs} = \frac{P_{HONO\,\mathrm{het}}}{j\mathrm{HNO_3} \times [\mathrm{pNO_3^-}]_{\mathrm{bulk}}} \quad \text{(XV)}$$

To determine the uncertainty in f_{obs}, the uncertainties were propagated using the rule for division making the average total uncertainty in the enhancement factor 57%.

9 ISORROPIA-II analyses: Partitioning of pNO_3^- and HNO_3

The thermodynamic equilibrium model ISORROPIA-II (*38*), used to investigate K^+–Ca^{2+}–Mg^{2+}–NH_4^+–Na^+–SO_4^{2-}–NO_3^-–Cl^-–H_2O aerosol systems, was utilised to estimate the partitioning of HNO_3 (g) and pNO_3^- during the aircraft campaigns. Calculations were performed in the forward mode in which the total (gas + aerosol) concentrations of the aforementioned species are specified alongside ambient temperatures and relative humidities, with the aerosol assumed to be in a thermodynamically stable state owing to the wide range of relative humidities experienced during the campaigns (5-96%). Consequently, the aerosol can either exist in the solid and/or liquid state during the calculations. From the above calculations, pNO_3^- was estimated to be the dominant fraction of the total nitrate measurements recorded, with no gaseous HNO_3 predicted in any of the deliquesced aerosol samples collected across ARNA.

SI figures and tables:

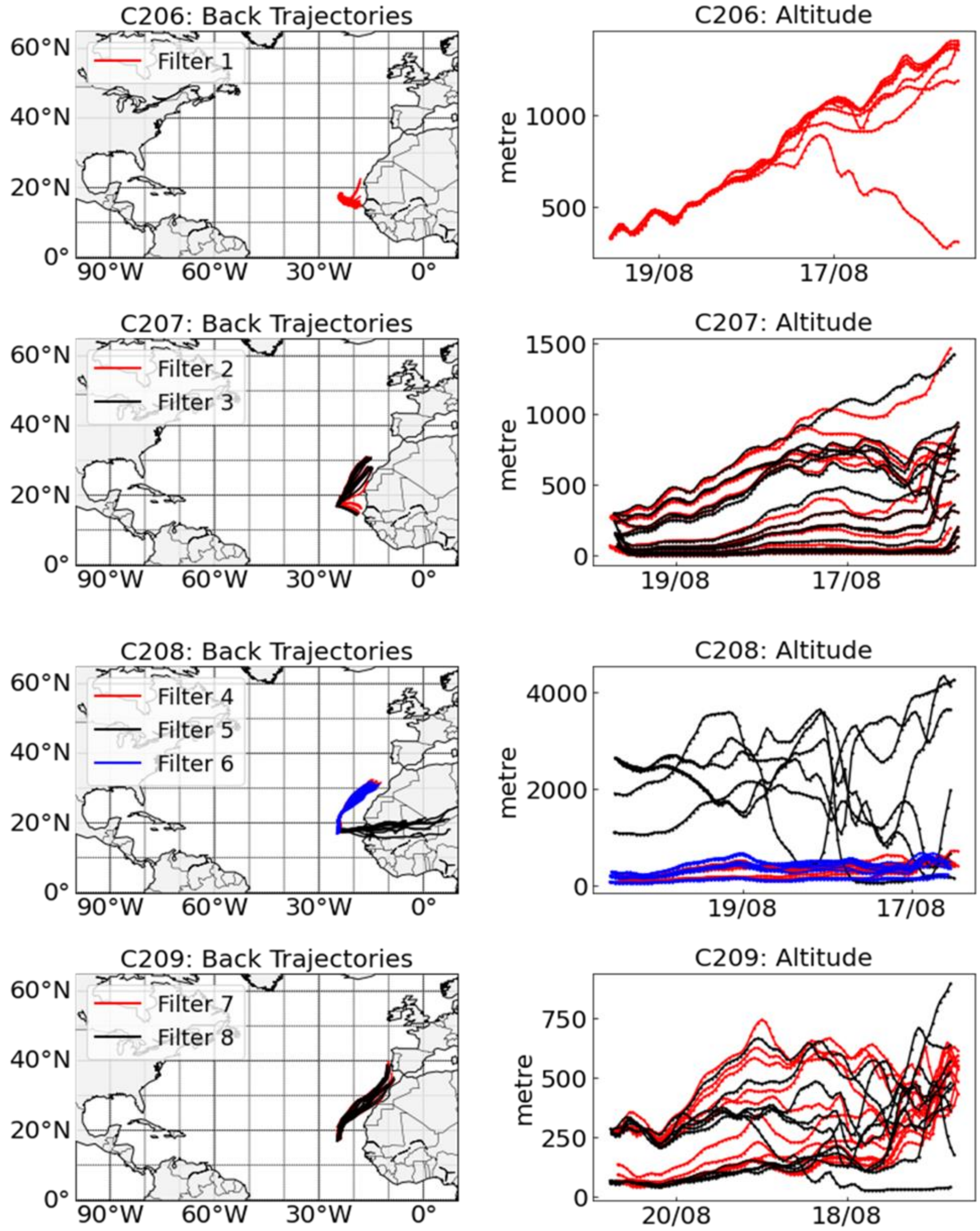

Fig. S1. 96 h HYSPLIT back trajectories for each aerosol sample during ARNA-1. All trajectories along one SLR are colored the same.

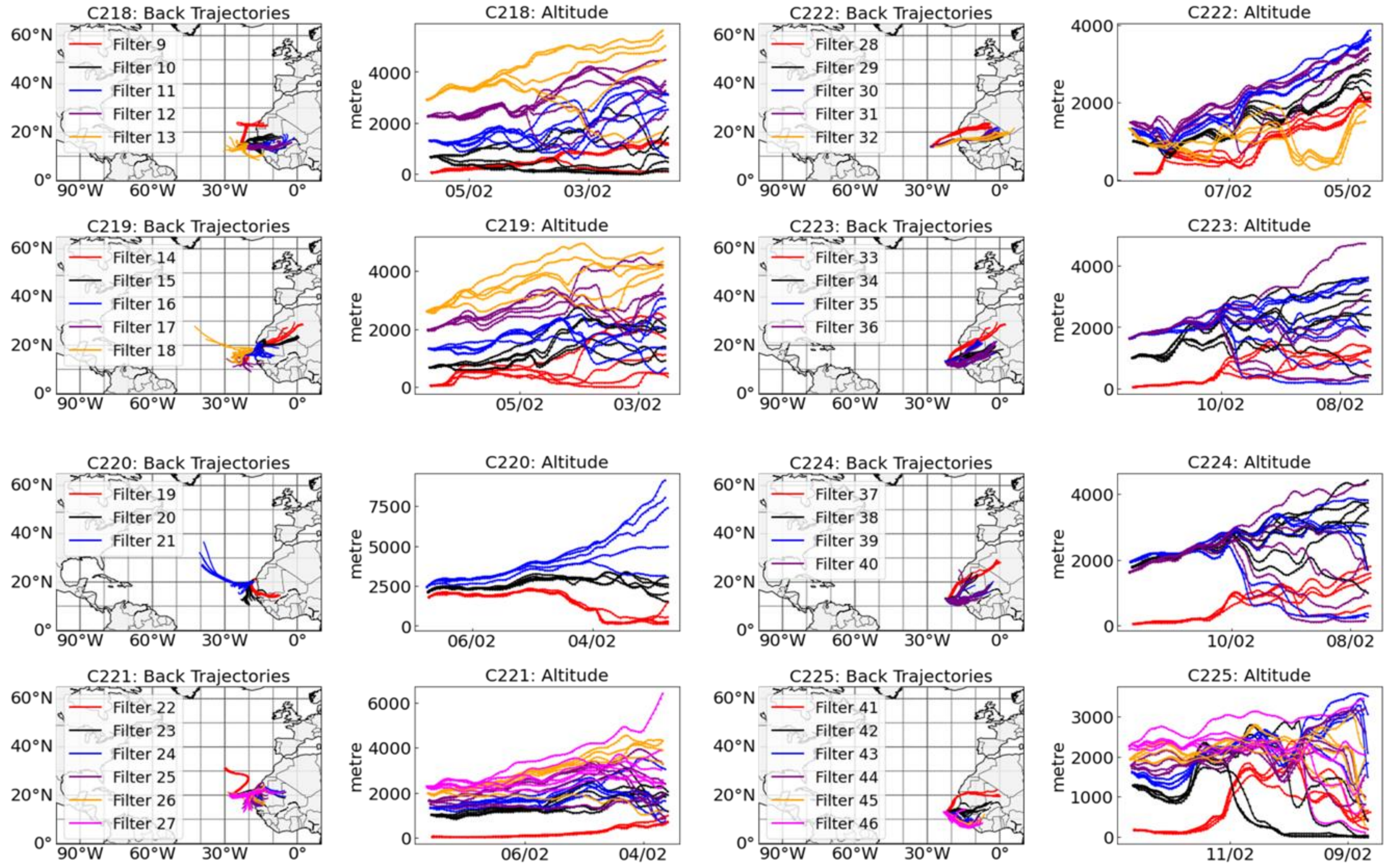

Fig. S2. 96 h HYSPLIT back trajectories for each aerosol sample during ARNA-2. All trajectories along one SLR are colored the same.

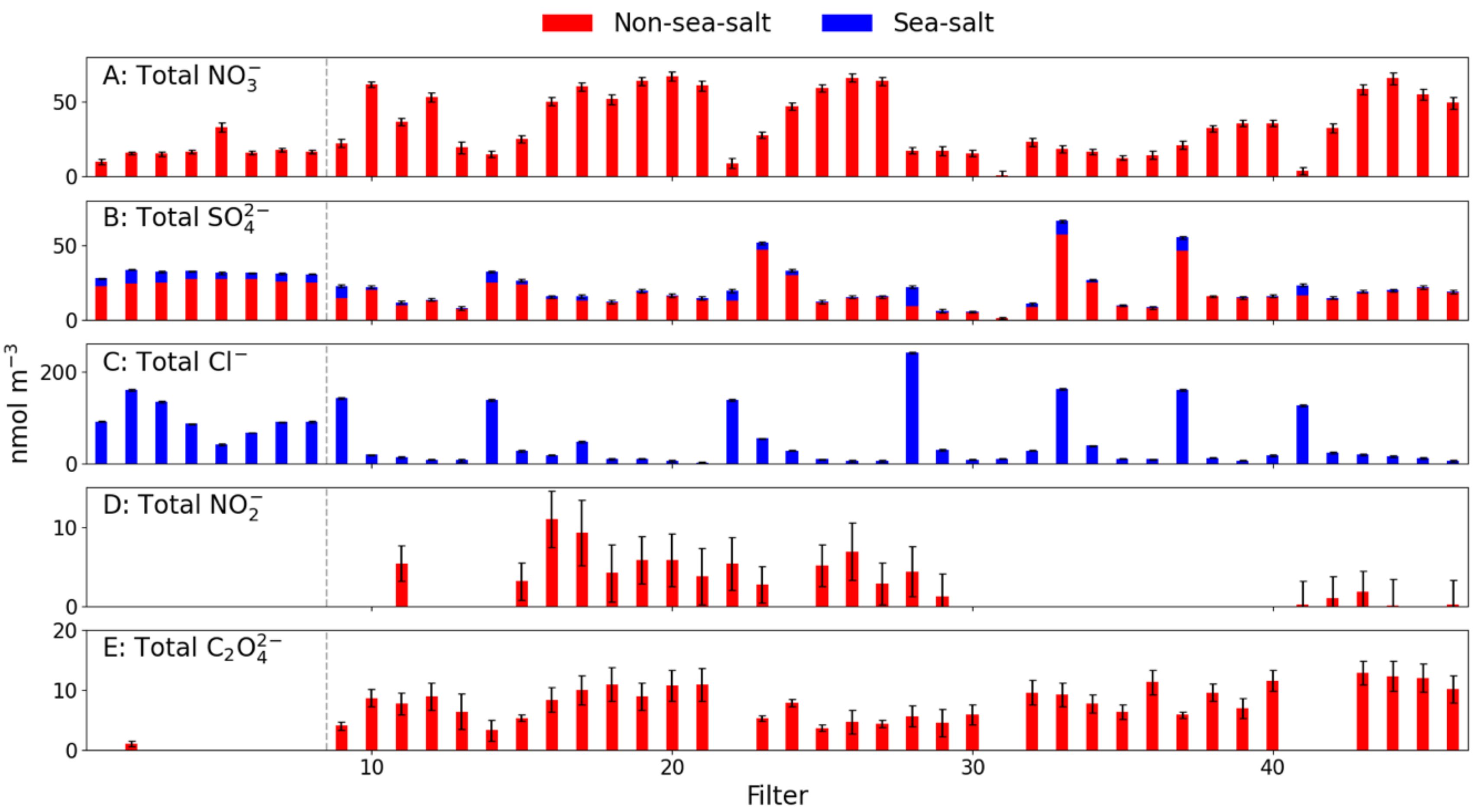

Fig. S3. Total concentrations (< 1 µm + > 1 µm) of all the anions measured for each filter during ARNA-1 (1-8) and ARNA-2 (9-46). The error bars symbolize the uncertainty of the total concentration and the grey dashed vertical lines separate the two campaigns.

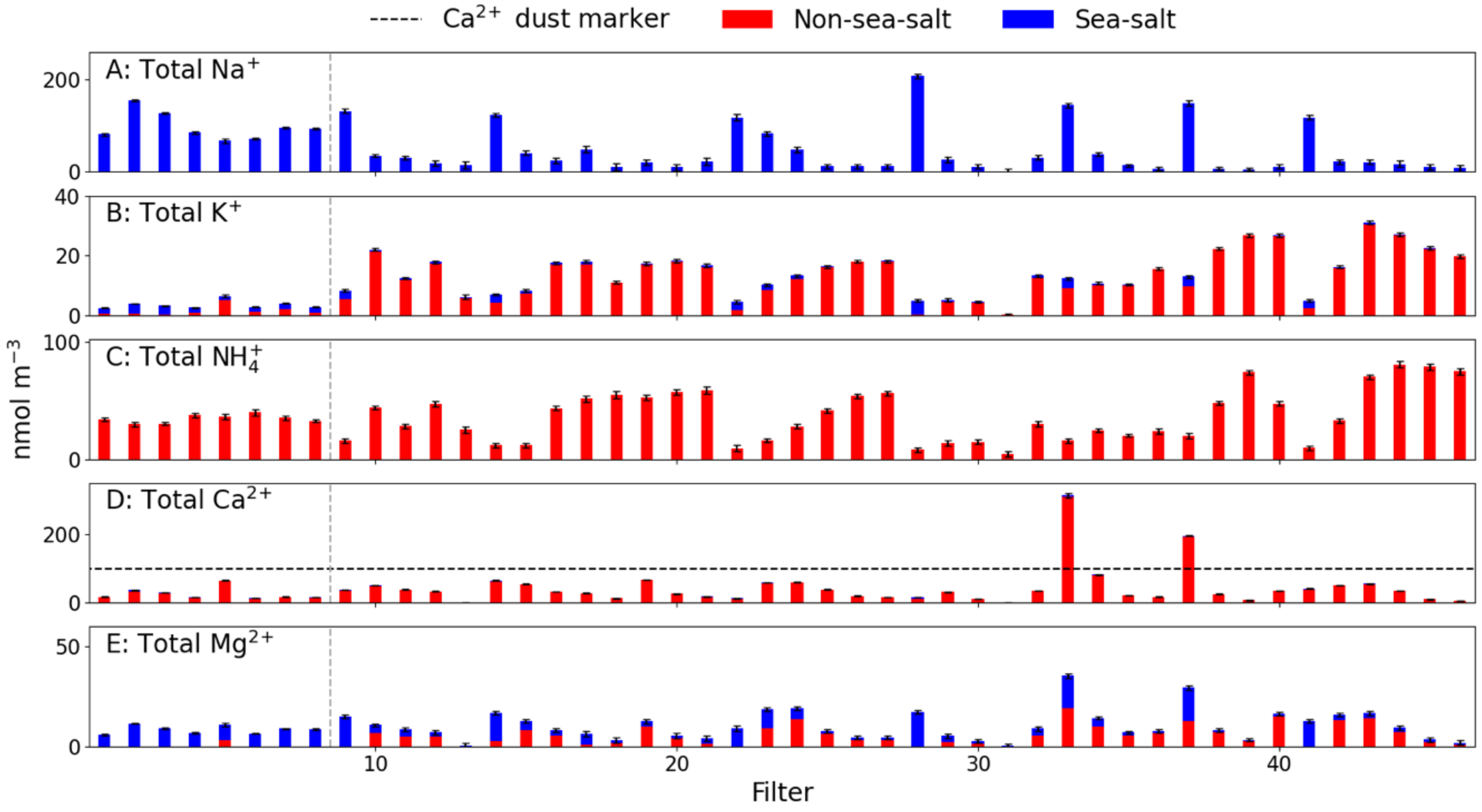

Fig. S4. Total concentrations (< 1 µm + > 1 µm) of all the cations measured for each filter during ARNA-1 (1-8) and ARNA-2 (9-46). The error bars symbolize the uncertainty of the total concentration, the dashed black line marks amount of Ca^{2+} needed for a sample to be categorized as dust and the grey dashed vertical lines separate the two campaigns.

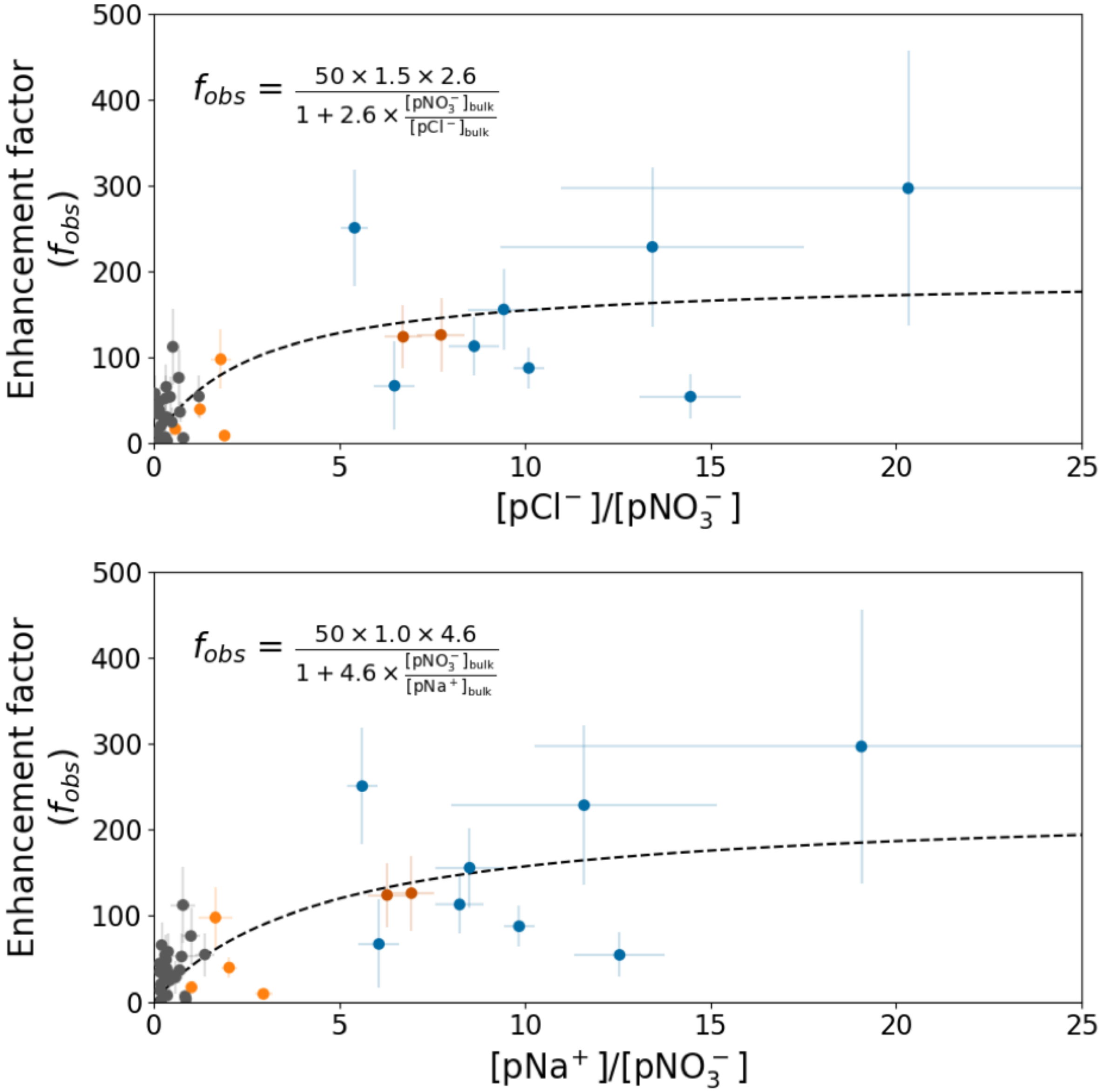

Fig. S5. Derived enhancement factor *f* as a function of [X]/ $[pNO_3^-]$ (molar concentration ratio), where X is pCl^- or pNa^+. The data points are colored by aerosol classification as in Fig. 3 and the dashed lines show the Langmuir isotherm fitted to the data according to the equations displayed in the respective panels.

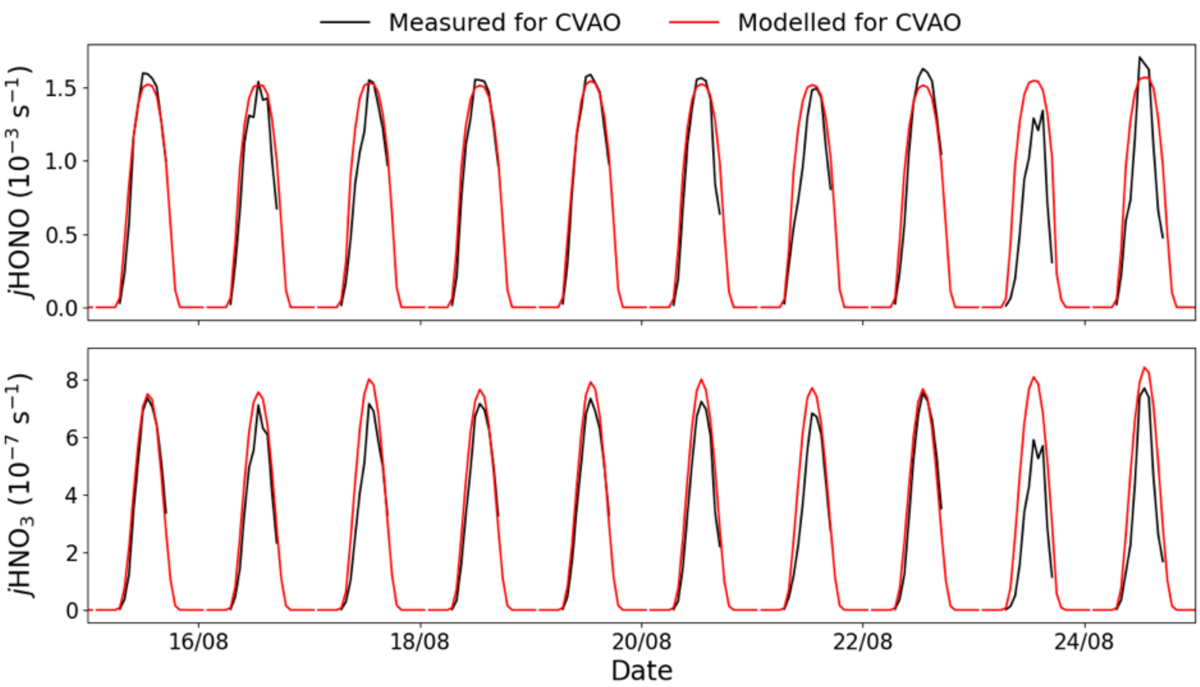

Fig. S6. Measured and modelled (GEOS-Chem) photolysis rates for HONO and HNO_3 at the CVAO in August 2019.

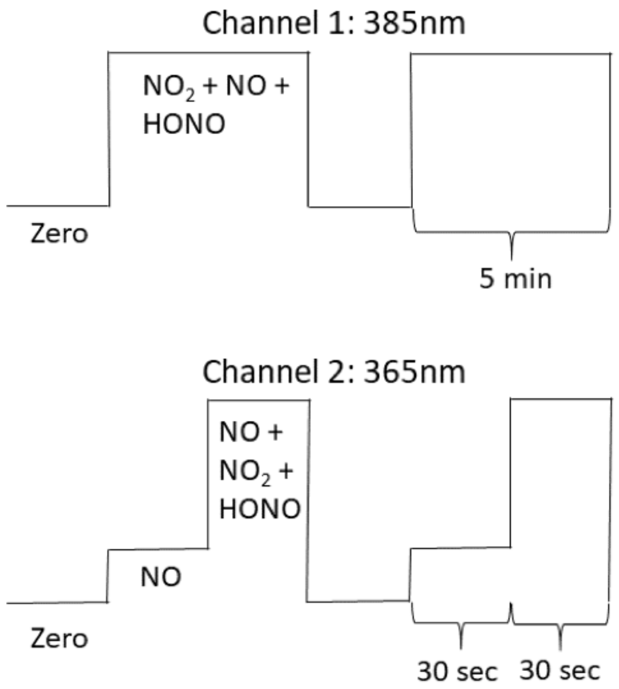

Fig. S7. Measurement cycles for the NO_x+HONO instrument on the FAAM Bae-146 aircraft.

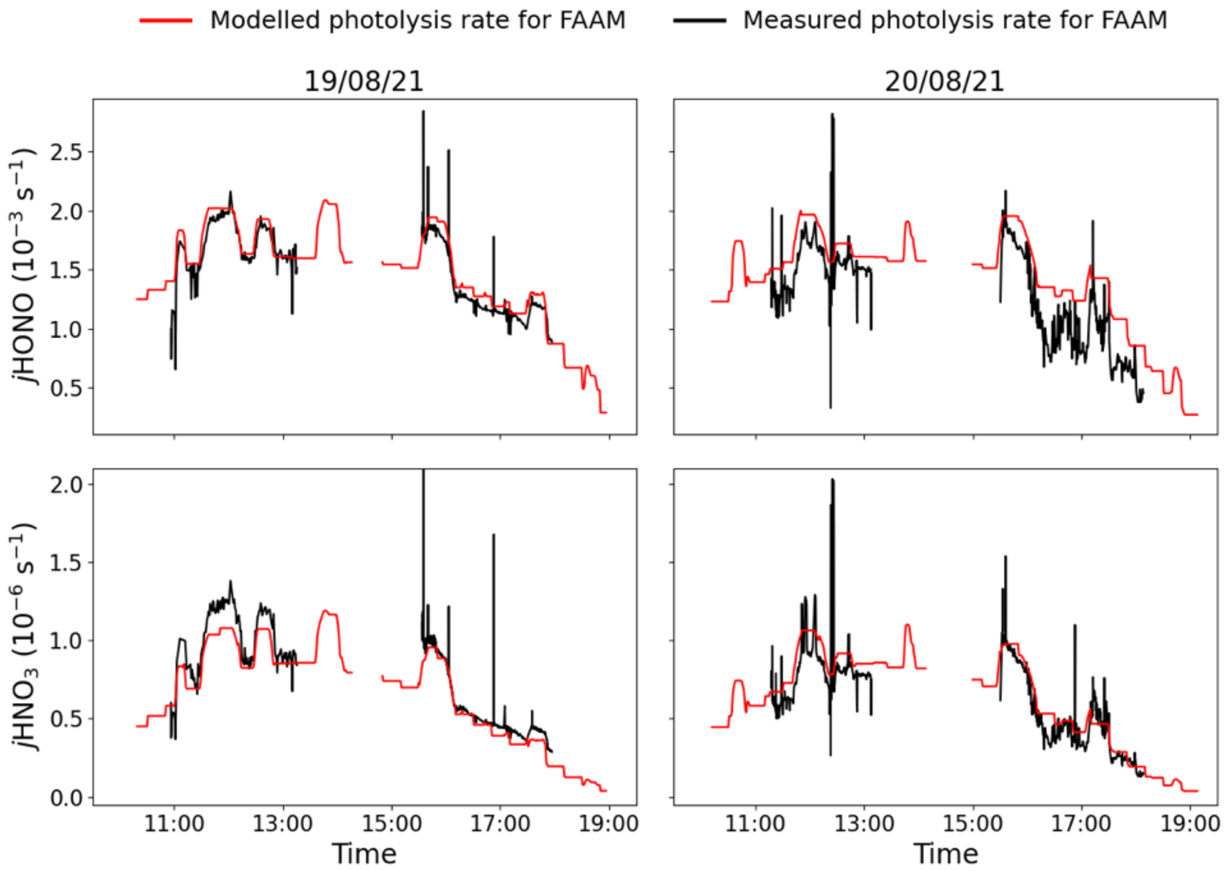

Fig. S8. Measured and modelled (GEOS-Chem) photolysis rates for HNO_3 and HONO aboard the FAAM Bae-146 aircraft. Two flights are plotted each day creating a gap in the middle of the measurements.

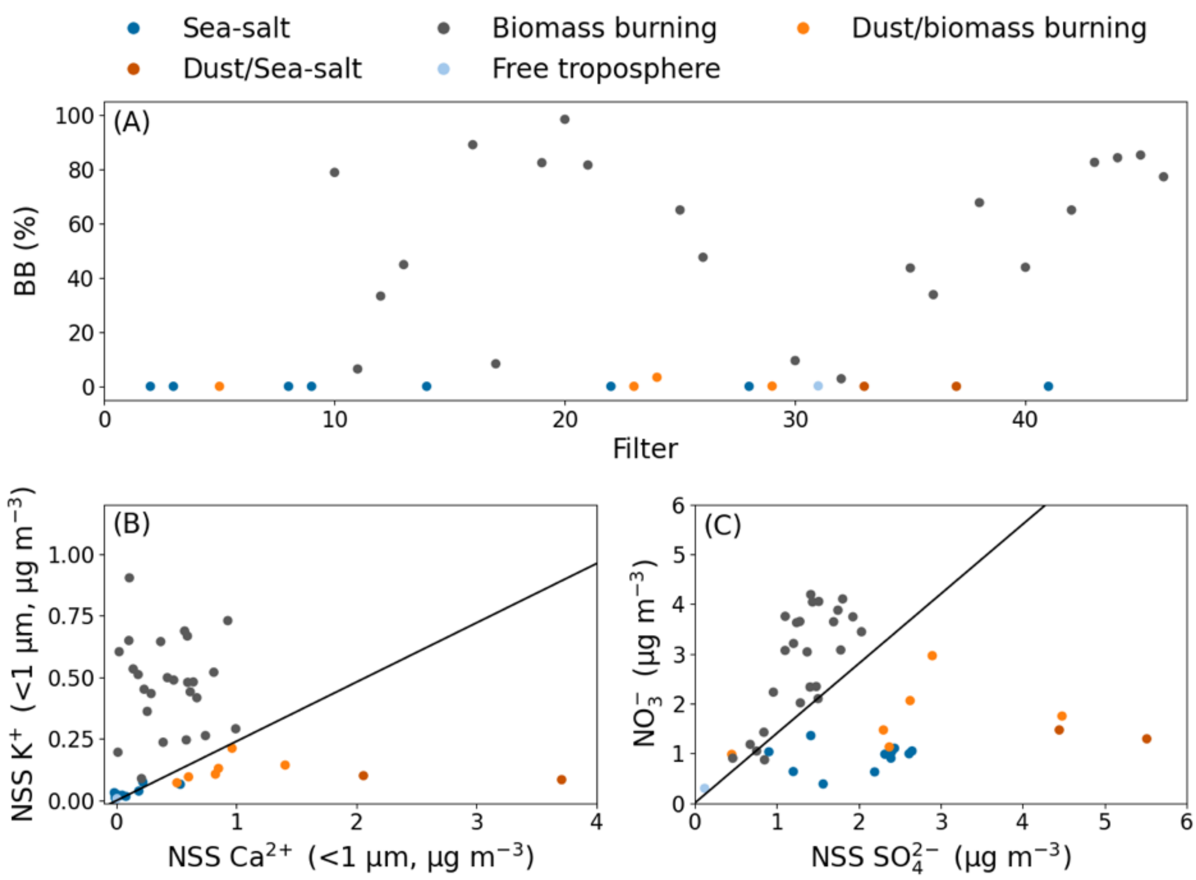

Fig. S9. Biomass burning tracers for each SLR. (A) shows the percentage of the sampling time spent in a biomass burning plume, defined according to the gas-phase biomass burning filter, for each aerosol sample. (B) shows the NSS K^+ as a function of the NSS Ca^{2+} in the fine size fraction, where the black line is the 0.24 ratio. (C) shows the total NO_3^- as a function of total NSS SO_4^{2-}, where the black line represents $NO_3^- = 1.4 \times$ NSS SO_4^{2-}. Each data point is colored by the determined air mass category based on aerosol composition, back trajectories, and the trace gases sampled.

Table S1. Comparison of aerosol composition during the ARNA campaigns, the SHADE campaign (*56*) and measurements made at the CVAO (*49*). The ARNA and CVAO data are given from min-max and the SHADE data is given as 25th-percentile-75th percentile. All concentrations are given in 10^{-9} moles/m^3.

	**ARNA-1 (Summer)**		**ARNA-2 (Winter)**		**SHADE[a] (*56*)**		**CVAO[b] (*49*)**
	< 1 µm	>1 µm	< 1 µm	> 1 µm	< 1 µm	> 1 µm	< 10 µm
**Cl^-**	7 - 13.5	31.4 - 153	Bdl - 30.8	Bdl - 210	1.3 - 3.6	1.9 - 31.3	9.9 - 597
**NO_3^-**	1.7 - 10.1	8.4 - 23.1	Bdl - 33.3	Bdl – 38.0	0.16 - 7.8	7.4 - 33.0	2.3 - 60.6
**SO_4^{2-}**	17.7 - 21.0	9.6 - 15.7	1.3 - 25.0	Bdl - 46.2	0.8 - 26.7	2.9 - 44.0	3.2 - 76.8
**NO_2^-**	Bdl	Bdl	Bdl - 5.1	Bdl - 8.2	-	-	-
**$C_2O_4^{2-}$**	Bdl - 1.0	Bdl	Bdl - 8.2	Bdl - 7.8	-	-	Bdl - 5.2
**Na^+**	Bdl - 17.1	35.7 - 146	Bdl - 34.5	Bdl - 179	6.9 - 14.6	6.5 - 75.7	10.9 - 554
**NH_4^+**	27.4 - 38.1	1.3 - 7.4	3.2 - 69.1	1.0 - 26.7	0.4 - 6.4	0.4 - 6.6	Bdl - 9.5
**K^+**	0.6 - 3.2	1.9 - 3.4	0.2 - 23.2	Bdl - 12.1	0.6 - 2.4	2.7 - 14.5	Bdl - 22.0
**Ca^{2+}**	Bdl - 15.7	12.5 - 49.3	Bdl - 93.1	Bdl - 221	2.5 - 15.9	19.7 - 196	Bdl - 110
**Mg^{2+}**	Bdl - 3.2	5.6 - 10.3	Bdl - 10.2	Bdl - 117	0.8 - 4.1	1.9 - 25.5	2.1 - 55.1

Bdl: Below detection limit

[a]All concentrations from the Saharan Dust Experiment (SHADE) have been converted from ng m^{-3} to nmol m^{-3}. Most samples were taken between 2.5 and 6 km above sea level.

[b]Measurements made at the CVAO between 2007 and 2011, which have been converted from µg m^{-3} to nmol m^{-3}

REFERENCES AND NOTES

1. P. S. Monks, C. Granier, S. Fuzzi, A. Stohl, M. L. Williams, H. Akimoto, M. Amann, A. Baklanov, U. Baltensperger, I. Bey, N. Blake, R. S. Blake, K. Carslaw, O. R. Cooper, F. Dentener, D. Fowler, E. Fragkou, G. J. Frost, S. Generoso, P. Ginoux, V. Grewe, A. Guenther, H. C. Hansson, S. Henne, J. Hjorth, A. Hofzumahaus, H. Huntrieser, I. S. A. Isaksen, M. E. Jenkin, J. Kaiser, M. Kanakidou, Z. Klimont, M. Kulmala, P. Laj, M. G. Lawrence, J. D. Lee, C. Liousse, M. Maione, G. McFiggans, A. Metzger, A. Mieville, N. Moussiopoulos, J. J. Orlando, C. D. O'Dowd, P. I. Palmer, D. D. Parrish, A. Petzold, U. Platt, U. Pöschl, A. S. H. Prévôt, C. E. Reeves, S. Reimann, Y. Rudich, K. Sellegri, R. Steinbrecher, D. Simpson, H. ten Brink, J. Theloke, G. R. van der Werf, R. Vautard, V. Vestreng, C. Vlachokostas, R. von Glasow, Atmospheric composition change—Global and regional air quality. *Atmos. Environ.* **43**, 5268–5350 (2009).

2. N. Theys, R. Volkamer, J. F. Müller, K. J. Zarzana, N. Kille, L. Clarisse, I. De Smedt, C. Lerot, H. Finkenzeller, F. Hendrick, T. K. Koenig, C. F. Lee, C. Knote, H. Yu, M. Van Roozendael, Global nitrous acid emissions and levels of regional oxidants enhanced by wildfires. *Nat. Geosci.* **13**, 681–686 (2020).

3. H. Su, Y. Cheng, R. Oswald, T. Behrendt, I. Trebs, F. X. Meixner, M. O. Andreae, P. Cheng, Y. Zhang, U. Pöschl, Soil nitrite as a source of atmospheric HONO and OH radicals. *Science* **333**, 1616–1618 (2011).

4. X. Lu, Y. Wang, J. Li, L. Shen, J. C. H. Fung, Evidence of heterogeneous HONO formation from aerosols and the regional photochemical impact of this HONO source. *Environ. Res. Lett.* **13**, 114002 (2018).

5. X. Li, F. Rohrer, A. Hofzumahaus, T. Brauers, R. Häseler, B. Bohn, S. Broch, H. Fuchs, S. Gomm, F. Holland, J. Jäger, J. Kaiser, F. N. Keutsch, I. Lohse, K. Lu, R. Tillmann, R. Wegener, G. M. Wolfe, T. F. Mentel, A. Kiendler-Scharr, A. Wahner, Missing gas-phase source of HONO inferred from Zeppelin measurements in the troposphere. *Science* **344**, 292–296 (2014).

6. N. Zhang, X. Zhou, P. B. Shepson, H. Gao, M. Alaghmand, B. Stirm, Aircraft measurement of HONO vertical profiles over a forested region. *Geophys. Res. Lett.* **36**, (2009).

7. Z. Liu, Y. Wang, F. Costabile, A. Amoroso, C. Zhao, L. G. Huey, R. Stickel, J. Liao, T. Zhu, Evidence of aerosols as a media for rapid daytime HONO production over China. *Environ. Sci. Technol.* **48**, 14386–14391 (2014).

8. L. R. Crilley, L. J. Kramer, F. D. Pope, C. Reed, J. D. Lee, L. J. Carpenter, L. D. J. Hollis, S. M. Ball, W. J. Bloss, Is the ocean surface a source of nitrous acid (HONO) in the marine boundary layer? *Atmos. Chem. Phys.* **21**, 18213–18225 (2021).

9. C. Ye, X. Zhou, D. Pu, J. Stutz, J. Festa, M. Spolaor, C. Tsai, C. Cantrell, R. L. Mauldin, T. Campos, A. Weinheimer, R. S. Hornbrook, E. C. Apel, A. Guenther, L. Kaser, B. Yuan, T. Karl, J. Haggerty, S. Hall, K. Ullmann, J. N. Smith, J. Ortega, C. Knote, Rapid cycling of reactive nitrogen in the marine boundary layer. *Nature* **532**, 489 (2016), 491.

10. C. Reed, M. J. Evans, L. R. Crilley, W. J. Bloss, T. Sherwen, K. A. Read, J. D. Lee, L. J. Carpenter, Evidence for renoxification in the tropical marine boundary layer. *Atmos. Chem. Phys.* **17**, 4081–4092 (2017).

11. J. N. Pitts, H. W. Biermann, A. M. Winer, E. C. Tuazon, Spectroscopic identification and measurement of gaseous nitrous acid in dilute auto exhaust. *Atmos. Environ.* **18**, 847–854 (1984).

12. Y. Zhu, Y. Wang, X. Zhou, Y. F. Elshorbany, C. Ye, M. Hayden, A. J. Peters, An investigation into the chemistry of HONO in the marine boundary layer at Tudor Hill Marine Atmospheric Observatory in Bermuda. *Atmos. Chem. Phys.* **22**, 6327–6346 (2022).

13. C. Zhu, B. Xiang, L. T. Chu, L. Zhu, 308 nm photolysis of nitric acid in the gas phase, on aluminum surfaces, and on ice films. *J. Phys. Chem. A* **114**, 2561–2568 (2010).

14. A. M. Baergen, D. J. Donaldson, Photochemical renoxification of nitric acid on real urban grime. *Environ. Sci. Technol.* **47**, 815–820 (2013).

15. J. Du, L. Zhu, Quantification of the absorption cross sections of surface-adsorbed nitric acid in the 335–365 nm region by Brewster angle cavity ring-down spectroscopy. *Chem. Phys. Lett.* **511**, 213–218 (2011).

16. N. K. Scharko, A. E. Berke, J. D. Raff, Release of nitrous acid and nitrogen dioxide from nitrate photolysis in acidic aqueous solutions. *Environ. Sci. Technol.* **48**, 11991–12001 (2014).

17. C. Ye, N. Zhang, H. Gao, X. Zhou, Photolysis of particulate nitrate as a source of HONO and NO_x. *Environ. Sci. Technol.* **51**, 6849–6856 (2017).

18. C. Ye, N. Zhang, H. Gao, X. Zhou, Matrix effect on surface-catalyzed photolysis of nitric acid. *Sci. Rep.* **9**, 4351 (2019).

19. X. Zhou, H. Gao, Y. He, G. Huang, S. B. Bertman, K. Civerolo, J. Schwab, Nitric acid photolysis on surfaces in low-NO_x environments: Significant atmospheric implications. *Geophys. Res. Lett.* **30**, (2003).

20. C. Ye, H. Gao, N. Zhang, X. Zhou, Photolysis of nitric acid and nitrate on natural and artificial surfaces. *Environ. Sci. Technol.* **50**, 3530–3536 (2016).

21. P. Kasibhatla, T. Sherwen, M. J. Evans, L. J. Carpenter, C. Reed, B. Alexander, Q. Chen, M. P. Sulprizio, J. D. Lee, K. A. Read, W. Bloss, L. R. Crilley, W. C. Keene, A. A. P. Pszenny, A. Hodzic, Global impact of nitrate photolysis in sea-salt aerosol on NO_x, OH, and O_3 in the marine boundary layer. *Atmos. Chem. Phys.* **18**, 11185–11203 (2018).

22. C. Ye, D. E. Heard, L. K. Whalley, Evaluation of novel routes for NO_x formation in remote regions. *Environ. Sci. Technol.* **51**, 7442–7449 (2017).

23. Q. Shi, Y. Tao, J. E. Krechmer, C. L. Heald, J. G. Murphy, J. H. Kroll, Q. Ye, Laboratory investigation of renoxification from the photolysis of inorganic particulate nitrate. *Environ. Sci. Technol.* **55**, 854–861 (2021).

24. P. S. Romer, P. J. Wooldridge, J. D. Crounse, M. J. Kim, P. O. Wennberg, J. E. Dibb, E. Scheuer, D. R. Blake, S. Meinardi, A. L. Brosius, A. B. Thames, D. O. Miller, W. H. Brune, S. R. Hall, T. B. Ryerson, R. C. Cohen, Constraints on aerosol nitrate photolysis as a potential source of HONO and NO_x. *Environ. Sci. Technol.* **52**, 13738–13746 (2018).

25. J. Zeng, G. Zhang, S. Long, K. Liu, L. Cao, L. Bao, Y. Li, Sea salt deliquescence and crystallization in atmosphere: An in situ investigation using x-ray phase contrast imaging. *Surf. Interface Anal.* **45**, 930–936 (2013).

26. S. A. Asher, D. D. Tuschel, T. A. Vargson, L. Wang, S. J. Geib, Solid state and solution nitrate photochemistry: Photochemical evolution of the solid state lattice. *Chem. A Eur. J.* **115**, 4279–4287 (2011).

27. C. Zhu, B. Xiang, L. Zhu, R. Cole, Determination of absorption cross sections of surface-adsorbed HNO_3 in the 290–330 nm region by Brewster angle cavity ring-down spectroscopy. *Chem. Phys. Lett.* **458**, 373–377 (2008).

28. J. Cheng, C. D. Vecitis, M. R. Hoffmann, A. J. Colussi, Experimental anion affinities for the air/water interface. *J. Phys. Chem. B.* **110**, 25598–25602 (2006).

29. M. Xu, R. Spinney, H. C. Allen, Water structure at the air−aqueous interface of divalent cation and nitrate solutions. *J. Phys. Chem. B.* **113**, 4102–4110 (2009).

30. W. Hua, D. Verreault, H. C. Allen, Surface electric fields of aqueous solutions of NH_4NO_3, $Mg(NO_3)_2$, $NaNO_3$, and $LiNO_3$: Implications for atmospheric aerosol chemistry. *J. Phys. Chem. C* **118**, 24941–24949 (2014).

31. L. M. Wingen, A. C. Moskun, S. N. Johnson, J. L. Thomas, M. Roeselová, D. J. Tobias, M. T. Kleinman, B. J. Finlayson-Pitts, Enhanced surface photochemistry in chloride–nitrate ion mixtures. *Phys. Chem. Chem. Phys.* **10**, 5668–5677 (2008).

32. N. K. Richards, B. J. Finlayson-Pitts, Production of gas phase NO_2 and halogens from the photochemical oxidation of aqueous mixtures of sea salt and nitrate Ions at room temperature. *Environ. Sci. Technol.* **46**, 10447–10454 (2012).

33. N. K. Richards, L. M. Wingen, K. M. Callahan, N. Nishino, M. T. Kleinman, D. J. Tobias, B. J. Finlayson-Pitts, Nitrate ion photolysis in thin water films in the presence of bromide ions. *J. Phys. Chem. A* **115**, 5810–5821 (2011).

34. R. Zhang, M. Gen, D. Huang, Y. Li, C. K. Chan, Enhanced sulfate production by nitrate photolysis in the presence of halide ions in atmospheric particles. *Environ. Sci. Technol.* **54**, 3831–3839 (2020).

35. A. C. Hong, S. N. Wren, D. J. Donaldson, Enhanced surface partitioning of nitrate anion in aqueous bromide solutions. *J. Phys. Chem. Lett.* **4**, 2994–2998 (2013).

36. S. R. Handley, D. Clifford, D. J. Donaldson, Photochemical loss of nitric acid on organic films: A possible recycling mechanism for NO_x. *Environ. Sci. Technol.* **41**, 3898–3903 (2007).

37. N. Nishino, S. A. Hollingsworth, A. C. Stern, M. Roeselová, D. J. Tobias, B. J. Finlayson-Pitts, Interactions of gaseous HNO_3 and water with individual and mixed alkyl self-assembled monolayers at room temperature. *Phys. Chem. Chem. Phys.* **16**, 2358–2367 (2014).

38. C. Fountoukis, A. Nenes, ISORROPIA II: A computationally efficient thermodynamic equilibrium model for K^+, Ca^{2+}, Mg^{2+}, NH_4^+, Na^+, SO_4^{2--}, NO_3^{--}, Cl^{--}, H_2O aerosols. *Atmos. Chem. Phys.* **7**, 4639–4659 (2007).

39. M. Gen, Z. Liang, R. Zhang, B. R. Go Mabato, C. K. Chan, Particulate nitrate photolysis in the atmosphere. *Environ. Sci. Atmos.* **2**, 111–127 (2022).

40. M. Roca, J. Zahardis, J. Bone, M. El-Maazawi, V. H. Grassian, 310 nm irradiation of atmospherically relevant concentrated aqueous nitrate solutions: Nitrite production and quantum yields. *J. Phys. Chem. A* **112**, 13275–13281 (2008).

41. M. Ndour, P. Conchon, B. D'Anna, O. Ka, C. George, Photochemistry of mineral dust surface as a potential atmospheric renoxification process. *Geophys. Res. Lett.* **36**, (2009).

42. D. I. Reeser, N.-O. A. Kwamena, D. J. Donaldson, Effect of organic coatings on gas-phase nitrogen dioxide production from aqueous nitrate photolysis. *J. Phys. Chem. C* **117**, 22260–22267 (2013).

43. J. E. Dyson, G. A. Boustead, L. T. Fleming, M. Blitz, D. Stone, S. R. Arnold, L. K. Whalley, D. E. Heard, Production of HONO from NO_2 uptake on illuminated TiO_2 aerosol particles and following the illumination of mixed TiO_2 ammonium nitrate particles. *Atmos. Chem. Phys.* **21**, 5755–5775 (2021).

44. S. L. Mora Garcia, S. Pandit, J. G. Navea, V. H. Grassian, Nitrous acid (HONO) formation from the irradiation of aqueous nitrate solutions in the presence of marine chromophoric dissolved organic matter: Comparison to other Organic photosensitizers. *ACS Earth Space Chem.* **5**, 3056–3064 (2021).

45. X. Wang, E. Z. Dalton, Z. C. Payne, S. Perrier, M. Riva, J. D. Raff, C. George, Superoxide and nitrous acid production from nitrate photolysis is enhanced by dissolved aliphatic organic matter. *Environ. Sci. Technol. Lett.* **8**, 53–58 (2021).

46. S. E. Bauer, D. Koch, N. Unger, S. M. Metzger, D. T. Shindell, D. G. Streets, Nitrate aerosols today and in 2030: A global simulation including aerosols and tropospheric ozone. *Atmos. Chem. Phys.* **7**, 5043–5059 (2007).

47. J. Heland, J. Kleffmann, R. Kurtenbach, P. Wiesen, A new instrument to measure gaseous nitrous acid (HONO) in the atmosphere. *Environ. Sci. Technol.* **35**, 3207–3212 (2001).

48. J. Kleffmann, P. Wiesen, Technical note: Quantification of interferences of wet chemical HONO LOPAP measurements under simulated polar conditions. *Atmos. Chem. Phys.* **8**, 6813–6822 (2008).

49. K. W. Fomba, K. Müller, D. van Pinxteren, L. Poulain, M. van Pinxteren, H. Herrmann, Long-term chemical characterization of tropical and marine aerosols at the Cape Verde Atmospheric Observatory (CVAO) from 2007 to 2011. *Atmos. Chem. Phys.* **14**, 8883–8904 (2014).

50. S. T. Andersen, L. J. Carpenter, B. S. Nelson, L. Neves, K. A. Read, C. Reed, M. Ward, M. J. Rowlinson, J. D. Lee, Long-term NO_x measurements in the remote marine tropical troposphere. *Atmos. Meas. Tech.* **14**, 3071–3085 (2021).

51. J. D. Lee, S. J. Moller, K. A. Read, A. C. Lewis, L. Mendes, L. J. Carpenter, Year-round measurements of nitrogen oxides and ozone in the tropical North Atlantic marine boundary layer. *J. Geophys. Res. Atmos.* **114**, (2009).

52. C. Reed, C. A. Brumby, L. R. Crilley, L. J. Kramer, W. J. Bloss, P. W. Seakins, J. D. Lee, L. J. Carpenter, HONO measurement by differential photolysis. *Atmos. Meas. Tech.* **9**, 2483–2495 (2016).

53. D. R. Glowacki, A. Goddard, K. Hemavibool, T. L. Malkin, R. Commane, F. Anderson, W. J. Bloss, D. E. Heard, T. Ingham, M. J. Pilling, P. W. Seakins, Design of and initial results from a highly instrumented reactor for atmospheric chemistry (HIRAC). *Atmos. Chem. Phys.* **7**, 5371–5390 (2007).

54. J. Mao, D. J. Jacob, M. J. Evans, J. R. Olson, X. Ren, W. H. Brune, J. M. S. Clair, J. D. Crounse, K. M. Spencer, M. R. Beaver, P. O. Wennberg, M. J. Cubison, J. L. Jimenez, A. Fried, P. Weibring, J. G. Walega, S. R. Hall, A. J. Weinheimer, R. C. Cohen, G. Chen, J. H. Crawford, C. McNaughton, A. D. Clarke, L. Jaeglé, J. A. Fisher, R. M. Yantosca, P. Le Sager, C. Carouge, Chemistry of hydrogen oxide radicals (HO_x) in the Arctic troposphere in spring. *Atmos. Chem. Phys.* **10**, 5823–5838 (2010).

55. H. Bian, M. J. Prather, Fast-J2: Accurate simulation of stratospheric photolysis in global chemical models. *J. Atmos. Chem.* **41**, 281–296 (2002).

56. P. Formenti, W. Elbert, W. Maenhaut, J. Haywood, M. O. Andreae, Chemical composition of mineral dust aerosol during the Saharan Dust Experiment (SHADE) airborne campaign in the Cape Verde region, September 2000. *J. Geophys. Res. Atmos.* **108**, 8576 (2003).

57. M. O. Andreae, W. Elbert, R. Gabriel, D. W. Johnson, S. Osborne, R. Wood, Soluble ion chemistry of the atmospheric aerosol and SO_2 concentrations over the eastern North Atlantic during ACE-2. *Tellus B* **52**, 1066–1087 (2000).

58. A. Sanchez-Marroquin, D. H. P. Hedges, M. Hiscock, S. T. Parker, P. D. Rosenberg, J. Trembath, R. Walshaw, I. T. Burke, J. B. McQuaid, B. J. Murray, Characterisation of the filter inlet system on the FAAM BAe-146 research aircraft and its use for size-resolved aerosol composition measurements. *Atmos. Meas. Tech.* **12**, 5741–5763 (2019).

59. L. J. Carpenter, Z. L. Fleming, K. A. Read, J. D. Lee, S. J. Moller, J. R. Hopkins, R. M. Purvis, A. C. Lewis, K. Müller, B. Heinold, H. Herrmann, K. W. Fomba, D. van Pinxteren, C. Müller, I. Tegen, A. Wiedensohler, T. Müller, N. Niedermeier, E. P. Achterberg, M. D. Patey, E. A. Kozlova, M. Heimann, D. E. Heard, J. M. C. Plane, A. Mahajan, H. Oetjen, T. Ingham, D. Stone, L. K. Whalley, M. J. Evans, M. J. Pilling, R. J. Leigh, P. S. Monks, A. Karunaharan, S. Vaughan, S. R. Arnold, J. Tschritter, D. Pöhler, U. Frieß, R. Holla, L. M. Mendes, H. Lopez, B. Faria, A. J. Manning, D. W. R. Wallace, Seasonal characteristics of tropical marine boundary layer air measured at the Cape Verde Atmospheric Observatory. *J. Atmos. Chem.* **67**, 87–140 (2010).

60. K. A. Read, A. S. Mahajan, L. J. Carpenter, M. J. Evans, B. V. E. Faria, D. E. Heard, J. R. Hopkins, J. D. Lee, S. J. Moller, A. C. Lewis, L. Mendes, J. B. McQuaid, H. Oetjen, A. Saiz-Lopez, M. J. Pilling, J. M. C. Plane, Extensive halogen-mediated ozone destruction over the tropical Atlantic Ocean. *Nature* **453**, 1232 (2008), 1235.

61. M. J. A. Rijkenberg, C. F. Powell, M. Dall'Osto, M. C. Nielsdottir, M. D. Patey, P. G. Hill, A. R. Baker, T. D. Jickells, R. M. Harrison, E. P. Achterberg, Changes in iron speciation following a Saharan dust event in the tropical North Atlantic Ocean. *Mar. Chem.* **110**, 56–67 (2008).

62. I. Chiapello, G. Bergametti, L. Gomes, B. Chatenet, F. Dulac, J. Pimenta, E. S. Suares, An additional low layer transport of Sahelian and Saharan dust over the north-eastern Tropical Atlantic. *Geophys. Res. Lett.* **22**, 3191–3194 (1995).

63. C. A. Keller, K. E. Knowland, B. N. Duncan, J. Liu, D. C. Anderson, S. Das, R. A. Lucchesi, E. W. Lundgren, J. M. Nicely, E. Nielsen, L. E. Ott, E. Saunders, S. A. Strode, P. A. Wales, D. J. Jacob, S. Pawson, Description of the NASA GEOS Composition Forecast Modeling System GEOS-CF v1.0. *J. Adv. Model. Earth Syst.* **13**, e2020MS002413 (2021).

64. I. B. Pollack, B. M. Lerner, T. B. Ryerson, Evaluation of ultraviolet light-emitting diodes for detection of atmospheric NO_2 by photolysis-chemiluminescence. *J. Atmos. Chem.* **65**, 111–125 (2010).

65. C. Reed, M. J. Evans, P. D. Carlo, J. D. Lee, L. J. Carpenter, Interferences in photolytic NO_2 measurements: Explanation for an apparent missing oxidant? *Atmos. Chem. Phys.* **16**, 4707–4724 (2016).

66. G. A. Boustead, "Measurement of nitrous acid production from aerosol surfaces using photo-fragmentation laser induced fluorescence," thesis, University of Leeds, (2019).

67. D. Silvia, J. Skilling, *Data Analysis: A Bayesian Tutorial* (Oxford Univ. Press, ed. 2, 2006).

68. R. Chance, T. D. Jickells, A. R. Baker, Atmospheric trace metal concentrations, solubility and deposition fluxes in remote marine air over the south-east Atlantic. *Mar. Chem.* **177**, 45–56 (2015).

69. M. O. Andreae, Soot carbon and excess fine potassium: Long-range transport of combustion-derived aerosols. *Science* **220**, 1148–1151 (1983).

70. A. R. Baker, T. D. Jickells, K. F. Biswas, K. Weston, M. French, Nutrients in atmospheric aerosol particles along the Atlantic Meridional Transect. *Deep-Sea Res. II Top. Stud. Oceanogr.* **53**, 1706–1719 (2006).

71. W. Stumm, J. J. Morgan, in *Aquatic Chemistry* (John Wiley, 1996), pp. 1022.

72. H. C. Price, K. J. Baustian, J. B. McQuaid, A. Blyth, K. N. Bower, T. Choularton, R. J. Cotton, Z. Cui, P. R. Field, M. Gallagher, R. Hawker, A. Merrington, A. Miltenberger, R. R. Neely III, S. T. Parker, P. D. Rosenberg, J. W. Taylor, J. Trembath, J. Vergara-Temprado, T.

F. Whale, T. W. Wilson, G. Young, B. J. Murray, Atmospheric ice-nucleating particles in the dusty tropical Atlantic. *J. Geophys. Res. Atmos.* **123**, 2175–2193 (2018).

73. P. D. Rosenberg, A. R. Dean, P. I. Williams, J. R. Dorsey, A. Minikin, M. A. Pickering, A. Petzold, Particle sizing calibration with refractive index correction for light scattering optical particle counters and impacts upon PCASP and CDP data collected during the Fennec campaign. *Atmos. Meas. Tech.* **5**, 1147–1163 (2012).

74. OPAC (Optical Properties of Aerosols and Clouds) aerosol database, https://geisa.aeris-data.fr/opac/ [accessed 4 June 2021].

75. M. Hess, P. Koepke, I. Schult, Optical properties of aerosols and clouds: The software package OPAC. *Bull. Am. Meteorol. Soc.* **79**, 831–844 (1998).

76. C. L. Ryder, F. Marenco, J. K. Brooke, V. Estelles, R. Cotton, P. Formenti, J. B. McQuaid, H. C. Price, D. Liu, P. Ausset, P. D. Rosenberg, J. W. Taylor, T. Choularton, K. Bower, H. Coe, M. Gallagher, J. Crosier, G. Lloyd, E. J. Highwood, B. J. Murray, Coarse-mode mineral dust size distributions, composition and optical properties from AER-D aircraft measurements over the tropical eastern Atlantic. *Atmos. Chem. Phys.* **18**, 17225–17257 (2018).

77. B. Weinzierl, D. Sauer, M. Esselborn, A. Petzold, A. Veira, M. Rose, S. Mund, M. Wirth, A. Ansmann, M. Tesche, S. Gross, V. Freudenthaler, Microphysical and optical properties of dust and tropical biomass burning aerosol layers in the Cape Verde region—An overview of the airborne in situ and lidar measurements during SAMUM-2. *Tellus B* **63**, 589–618 (2011).

78. C. L. Ryder, E. J. Highwood, A. Walser, P. Seibert, A. Philipp, B. Weinzierl, Coarse and giant particles are ubiquitous in Saharan dust export regions and are radiatively significant over the Sahara. *Atmos. Chem. Phys.* **19**, 15353–15376 (2019).

79. K. Kandler, K. Lieke, N. Benker, C. Emmel, M. Küpper, D. Müller-Ebert, M. Ebert, D. Scheuvens, A. Schladitz, L. Schütz, S. Weinbruch, Electron microscopy of particles collected

at Praia, Cape Verde, during the Saharan Mineral Dust Experiment: Particle chemistry, shape, mixing state and complex refractive index. *Tellus B* **63**, 475–496 (2011).

80. T. Müller, A. Schladitz, K. Kandler, A. Wiedensohler, Spectral particle absorption coefficients, single scattering albedos and imaginary parts of refractive indices from ground based in situ measurements at Cape Verde Island during SAMUM-2. *Tellus B* **63**, 573–588 (2011).

81. B. T. Johnson, S. R. Osborne, J. M. Haywood, M. A. J. Harrison, Aircraft measurements of biomass burning aerosol over West Africa during DABEX. *J. Geophys. Res. Atmos.* **113**, (2008).

82. K. Lieke, K. Kandler, D. Scheuvens, C. Emmel, C. V. Glahn, A. Petzold, B. Weinzierl, A. Veira, M. Ebert, S. Weinbruch, L. Schütz, Particle chemical properties in the vertical column based on aircraft observations in the vicinity of Cape Verde Islands. *Tellus B* **63**, 497–511 (2011).

83. S. Lance, C. A. Brock, D. Rogers, J. A. Gordon, Water droplet calibration of the Cloud Droplet Probe (CDP) and in-flight performance in liquid, ice and mixed-phase clouds during ARCPAC. *Atmos. Meas. Tech.* **3**, 1683–1706 (2010).

84. W. C. Hinds, *Aerosol Technology: Properties, Behavior, and Measurement of Airborne Particles* (Wiley, ed. 2, 1999).

85. K. R. Travis, C. L. Heald, H. M. Allen, E. C. Apel, S. R. Arnold, D. R. Blake, W. H. Brune, X. Chen, R. Commane, J. D. Crounse, B. C. Daube, G. S. Diskin, J. W. Elkins, M. J. Evans, S. R. Hall, E. J. Hintsa, R. S. Hornbrook, P. S. Kasibhatla, M. J. Kim, G. Luo, K. McKain, D. B. Millet, F. L. Moore, J. Peischl, T. B. Ryerson, T. Sherwen, A. B. Thames, K. Ullmann, X. Wang, P. O. Wennberg, G. M. Wolfe, F. Yu, Constraining remote oxidation capacity with ATom observations. *Atmos. Chem. Phys.* **20**, 7753–7781 (2020).

86. R. R. Draxler, G. Hess, Description of the HYSPLIT4 modeling system (Air Resources Laboratory, 1997).

87. J. D. Lee, F. A. Squires, T. Sherwen, S. E. Wilde, S. J. Cliff, L. J. Carpenter, J. R. Hopkins, S. J. Bauguitte, C. Reed, P. Barker, G. Allen, T. J. Bannan, E. Matthews, A. Mehra, C. Percival, D. E. Heard, L. K. Whalley, G. V. Ronnie, S. Seldon, T. Ingham, C. A. Keller, K. E. Knowland, E. G. Nisbet, S. Andrews, Ozone production and precursor emission from wildfires in Africa. *Environ. Sci. Atmos.* **1**, 524–542 (2021).

88. D. L. Savoie, J. M. Prospero, E. S. Saltzman, Non-sea-salt sulfate and nitrate in trade wind aerosols at Barbados: Evidence for long-range transport. *J. Geophys. Res. Atmos.* **94**, 5069–5080 (1989).

89. B. Mason, *Principles of Geochemistry* (Wiley, ed. 3, 1966).

90. R. Schlitzer, E. Masferrer Dodas, M. Adjou, R. F. Anderson, F. Andre, D. M. Cockwell, C. Jeandel, W. Geibert, W. Landing, M. Lohan, M. T. Maldonado, A. Tagliabue, W. Abouchami, E. P. Achterberg, A. M. Agather, A. Aguilar-Islas, H. Amakawa, P. Andersson, A. Annett, C. Archer, K. Arrigo, L. Artigue, M. Auro, O. Baars, I. Baconnais, A. Baker, K. Bakker, W. Bam, H. W. Bange, K. Barbeau, C. Basak, M. Baskaran, N. R. Bates, D. Bauch, F. Baurand, S. M. Becker, P. van Beek, M. Behrens, M. Belhadj, M. Belton, B. Bergquist, E. Black, S. Blain, K. Bluhm, A. R. Bowie, K. L. Bowman, P. Boyd, M. Boye, E. A. Boyle, P. Branellec, L. Bridgestock, G. Brissebrat, B. K. A., T. J. Browning, K. W. Bruland, M. Brzezinski, K. N. Buck, N. J. Buck, C. Buck, K. Buesseler, A. J. Bull, R. Bundy, E. Butler, P. H. Cai, D. Cardinal, C. Carlson, N. Casacuberta Arola, K. Casciotti, M. Castrillejo, E. Chamizo Calvo, R. Chance, M. A. Charette, B. L. A. Charlier, Z. Chase, J. E. Chavez, M. Cheize, M. Chen, H. Cheng, F. Chever, V. Chinni, R. Chmiel, M. Christl, T. M. Church, I. Closset, A. Colman, M. Colombo, M. Conte, T. M. Conway, M. Corkill, D. Cossa, S. E. Cravatte, K. C. Crocket, P. Croot, J. Cullen, G. A. Cutter, H. J. de Baar, T. van de Flierdt, G. de Souza, J. S. De Vera, F. Dehairs, F. Deng, P. van der Merwe, K. Djaoudi, A. Dufour, G. Dulaquais, Y. Echegoyen Sanz, L. Edwards, C. Ehlert, M. Ellwood, S. Fan, R. A. Fine, J. N. Fitzsimmons, M. Q. Fleisher, M. S. Forbes, R. Francois, M. Frank, J. Friedrich, F. Fripiat, H. Fröllje, J. Gagnon, S. J. G. Galer, M. Gallinari, T. Gamo, Y. Gao, J. García Orellana, E. Garcia Solsona, M. Gault-Ringold, E. George, L. J. A. Gerringa, M. Gilbert, J. M. Godoy, M. Le Goff, S. L. Goldstein, A. Gourain, J. Granger, P. Grasse, M. Grenier, E. Grossteffan, C.

Guieu, M. Gutjahr, R. Van Hale, C. R. Hammerschmidt, J. Happell, C. Hassler, E. C. Hathorne, M. Hatta, N. J. Hawco, C. T. Hayes, L. Heimburger, M. I. Heller, P. B. Henderson, G. Henderson, T. Henry, S. van Heuven, C. Holmden, T. M. Holmes, J. Hopkins, C. J. M. Hoppe, M. J. Hopwood, T. J. Horner, Y. Hsieh, K. Huang, M. P. Humphreys, S. L. Jackson, D. J. Janssen, W. J. Jenkins, L. T. Jensen, S. John, J. L. Jones, D. Kadko, R. Kayser, T. C. Kenna, J. Kenyon, R. Khondoker, D. Kieke, M. Kienast, T. Kim, L. Kipp, J. K. Klar, M. Klunder, Y. Kondo, K. Kreissig, S. Kretschmer, S. Krisch, K. Krupp, K. Kulinski, Y. Kumamoto, K. Kunde, P. Laan, F. Lacan, M. Lagarde, P. J. Lam, M. Lambelet, C. Lamborg, D. Lannuzel, P. Latour, G. Laukert, E. C. Laurenceau-Cornec, F. A. C. le Moigne, J. van Ooijen, E. Le Roy, E. van Weerlee, O. Lechtenfeld, J. Lee, N. Lehmann, N. Lemaitre, P. Lherminier, X. Li, J. Li, B. T. Liguori Pires, S. H. Little, M. Lopez Lora, D. E. Lott, A. J. M. Lough, Y. Lu, C. Mahaffey, K. Maiti, F. Marin, C. Marsay, P. Masqué, E. Mawji, M. R. McIlvin, C. Measures, S. Mehic, S. Michael, R. Middag, A. Miere, A. Milne, H. Minami, J. Moffett, W. Moore, B. Moran, P. Morton, A. Mucci, P. Mukherjee, J. Nishioka, R. L. Nixon, T. Noble, M. G. Novak, S. H. Nunige, H. Obata, H. Ogawa, D. C. Ohnemus, K. Orians, S. Ossebaar, J. E. O'Sullivan, K. Pahnke, M. Paul, F. J. Pavia, H. Perez-Tribouillier, M. M. G. Perron, B. D. Peters, M. V. Petrova, V. Pham, P. Pinedo-Gonzalez, A. Piotrowski, Y. Plancherel, F. Planchon, H. Planquette, A. Plante, S. Pohle, D. Porcelli, C. Pradoux, B. C. Proemse, V. Puigcorbe, E. Pulido-Villena, P. Quay, P. Rafter, I. Rapp, L. Ratnarajah, S. Rauschenberg, C. Rees, M. Rehkamper, R. Rember, T. Remenyi, J. A. Resing, S. Retelletti Brogi, M. J. A. Rijkenberg, L. F. Robinson, M. Roca Marti, T. Roeske, S. Roig, J. Rolison, M. Rosenberg, A. R. S. Ross, M. Roy-Barman, A. Ruacho, M. M. Rutgers van der Loeff, M. A. Saito, P. Salaun, V. Sanial, A. Santoro, G. Sarthou, C. Schallenberg, U. Schauer, H. Scher, C. Schlosser, N. Schuback, C. Schwanger, P. Scott, P. N. Sedwick, S. Selzer, K. Seyitmuhammedov, R. Shelley, R. Sherrell, K. Sherrin, A. M. Shiller, M. Sieber, D. Sigman, S. K. Singh, N. D. Singh, W. M. Smethie, A. J. R. Smith, Y. Sohrin, B. M. Sohst, J. E. Sonke, M. Souhaut, S. Speich, R. Steinfeldt, T. Stichel, I. Stimac, C. Stirling, B. Summers, G. J. Swarr, J. H. Swift, V. Taillandier, M. Thomas, S. Tibben, R. Till, A. T. Townsend, P. Tréguer, J. Tremblay, T. Trull, R. Tuerena, B. Twining, O. Valk, D. Vance, C. Venchiarutti, L. H. Vieira, M. Villa Alfageme, S. M. Vivancos, A. H. L. Voelker, T. Wagener, E. Wallner Halewood, R. Wang, F. Wang, R. Wang, M. J. Warner, R. J. Watson, T. Weber, A. Wefing,

R. Weisend, D. Weiss, L. M. Whitmore, K. Wong, E. M. S. Woodward, Y. Wu, J. Wu, K. Wuttig, N. J. Wyatt, Y. Xiang, R. C. Xie, S. Yu, P. Zhang, J. Zhang, L. Zheng, X. Zheng, C. M. Zurbrick, The GEOTRACES Intermediate Data Product 2021 (GEOTRACES, 2021).

91. R. Atkinson, D. L. Baulch, R. A. Cox, J. N. Crowley, R. F. Hampson, R. G. Hynes, M. E. Jenkin, M. J. Rossi, J. Troe, Evaluated kinetic and photochemical data for atmospheric chemistry: Volume I—Gas phase reactions of O_x, HO_x, NO_x and SO_x species. *Atmos. Chem. Phys.* **4**, 1461–1738 (2004).

92. R. M. Harrison, A.-M. N. Kitto, Evidence for a surface source of atmospheric nitrous acid. *Atmos. Environ.* **28**, 1089–1094 (1994).

93. R. M. Harrison, J. D. Peak, G. M. Collins, Tropospheric cycle of nitrous acid. *J. Geophys. Res. Atmos.* **101**, 14429–14439 (1996).

94. I. Trebs, L. L. Lara, L. M. M. Zeri, L. V. Gatti, P. Artaxo, R. Dlugi, J. Slanina, M. O. Andreae, F. X. Meixner, Dry and wet deposition of inorganic nitrogen compounds to a tropical pasture site (Rondônia, Brazil). *Atmos. Chem. Phys.* **6**, 447–469 (2006).

95. J. Stutz, B. Alicke, A. Neftel, Nitrous acid formation in the urban atmosphere: Gradient measurements of NO_2 and HONO over grass in Milan, Italy. *J. Geophys. Res. Atmos.* **107**, LOP 5-1–LOP 5-15 (2002).

96. J. W. Deardorff, Convective velocity and temperature scales for the unstable planetary boundary layer and for Rayleigh convection. *J. Atmos. Sci.* **27**, 1211–1213 (1970).

97. J. Kleffmann, Daytime sources of nitrous acid (HONO) in the atmospheric boundary layer. *ChemPhysChem* **8**, 1137–1144 (2007).

98. V. Michoud, A. Colomb, A. Borbon, K. Miet, M. Beekmann, M. Camredon, B. Aumont, S. Perrier, P. Zapf, G. Siour, W. Ait-Helal, C. Afif, A. Kukui, M. Furger, J. C. Dupont, M. Haeffelin, J. F. Doussin, Study of the unknown HONO daytime source at a European suburban site during the MEGAPOLI summer and winter field campaigns. *Atmos. Chem. Phys.* **14**, 2805–2822 (2014).

99. F. Spataro, A. Ianniello, Sources of atmospheric nitrous acid: State of the science, current research needs, and future prospects. *J. Air Waste Manage. Assoc.* **64**, 1232–1250 (2014).

100. M. Li, H. Su, G. Li, N. Ma, U. Pöschl, Y. Cheng, Relative importance of gas uptake on aerosol and ground surfaces characterized by equivalent uptake coefficients. *Atmos. Chem. Phys.* **19**, 10981–11011 (2019).
